# Identification of a specialized lipid barrier for *Drosophila* metamorphosis

**DOI:** 10.1126/sciadv.adw6168

**Published:** 2026-11-06

**Authors:** Lena Lampe, Clare L. Newell, Bing-Jun Wang, Andrew P. Bailey, Rami Makki, Cyrille Alexandre, Ian S. Gilmore, Li Zhao, Alex P. Gould

**Affiliations:** ^1^ Physiology and Metabolism Laboratory, The Francis Crick Institute, London NW1 1AT, UK.; ^2^ National Centre of Excellence in Mass Spectrometry Imaging, National Physical Laboratory, Teddington TW11 0LW, UK.; ^3^ Laboratory of Evolutionary Genetics and Genomics, The Rockefeller University, New York, NY 10065, USA.; ^4^ Epithelial Cell Interactions Laboratory, The Francis Crick Institute, London NW1 1AT, UK.

## Abstract

Many terrestrial insects transition from humid to dry environments at metamorphosis, yet how they avoid dehydration is unclear. In *Drosophila melanogaster*, we identify a lipid barrier to water loss in the pupa that is anatomically distinct from that of the adult. It comprises unisex hydrocarbons that are longer and less volatile than their sex-specific adult counterparts. These hyperlong hydrocarbons are produced by larval oenocytes and stored in the fat body for subsequent transport to the cuticle at pupariation. A fatty acid elongase, EloHL, is required for hyperlong hydrocarbon biosynthesis and for a functional barrier to dehydration during early metamorphosis. Hyperlong barrier hydrocarbons are widespread across the *Drosophila* genus and typically transition to shorter sex-specific versions during adult maturation. In the desert species *D. mojavensis*, however, adults retain hyperlong hydrocarbons. This study reveals how insect lipid barriers are tuned to environmental variations in aridity and temperature during development and evolution.

## INTRODUCTION

Dehydration is a potential threat to all terrestrial life. The evolution of multiple water conservation mechanisms has nevertheless allowed animals to colonize much of the earth’s land surface, including hot and arid zones (*1*, *2*). One critical adaptation for restricting water loss is the cutaneous lipid barrier—a hydrophobic coating of the integument that provides a direct interface between the animal and its environment. This widespread if not universal feature of terrestrial fauna is particularly important for small animals, like insects, as their large surface area to volume ratio makes them vulnerable to desiccation (*3*–*5*). Insects have therefore evolved very efficient cuticular lipid barriers to water loss, contributing to their remarkable success as the most diverse and species-rich class of land animals (*6*–*12*). The physicochemical properties and superficial location of insect cuticular lipids make them well suited to perform an additional function as semivolatile pheromones, conveying social cues about location and physiological status (*13*–*15*). The potential dual role of some cuticular lipids —as both a desiccation barrier and a pheromone—raises intriguing evolutionary questions about the links between ecological adaptation and speciation (*16*–*19*).

Holometabolous insects undergo complete metamorphosis. This remarkable and complex developmental process involves a marked morphological and physiological transformation of the larva into the adult (imago) and is often accompanied by a shift in ecological niche. In many holometabolous insects, including the fruit fly *Drosophila melanogaster*, larval stages typically burrow in a semiaquatic environment of rotting fruit but subsequent pupal and adult stages switch to a drier terrestrial environment that requires a more efficient barrier to desiccation (*20*, *21*). In adult insects, it is well established that their cuticles are coated with a lipid layer consisting primarily of hydrocarbons (*22*–*24*). These hydrocarbons play key roles both in desiccation resistance and in social communication, especially mating behavior (*17*, *25*–*27*). *Drosophila* hydrocarbons fall into four main categories—*n*-alkanes, methyl-branched alkanes, monoenes, and dienes. The precise blend of these determines the overall biophysical properties of the cuticular lipid barrier, such as its melting temperature, transpiration rate, and volatility (*28*–*31*). Adult oenocytes are the site of cuticular hydrocarbon synthesis in pterygote insects (*32*–*36*). In *D. melanogaster* and many other insects, these specialized lipid-secreting cells are confined to abdominal segments, where they are located beneath the epidermis and cross-talk with other tissues to coordinate lipid metabolism (*37*–*40*). Adult oenocytes synthesize hydrocarbons from fatty acyl–coenzyme A (CoA) precursors via reduction to fatty aldehydes and loss of one carbon by cytochrome P450–dependent oxidative decarbonylation (*41*, *42*). In *Drosophila*, knockout or oenocyte-specific knockdown of the P450 decarbonylase, Cyp4g1, results in loss of cuticular hydrocarbons, susceptibility to desiccation, and reduced viability upon adult emergence (eclosion) (*42*, *43*). Multiple fatty acid synthases, elongases, and desaturases expressed in adult oenocytes regulate the types of fatty acyl-CoA substrates available to Cyp4g1, in turn determining the length and desaturation of molecules in the complex blend of cuticular hydrocarbons (*44*–*49*).

Across the *Drosophila* genus, adult cuticular hydrocarbon profiles are highly divergent and tend to be sex specific (*31*, *50*). Natural selection is thought to favor the production of long-chain hydrocarbons with low volatility as these contribute most to an effective adult desiccation barrier (*30*, *51*–*53*). Consistent with this, carbon chain length correlates across species of *Drosophila*, and ants, with the temperature and humidity of their habitats (*54*–*56*). In particular, within the *Drosophila* genus, there is a strong correlation between the evolution of longer-chain methyl-branched hydrocarbons and higher desiccation resistance (*53*). The pheromonal role of adult hydrocarbons also places them under strong sexual selection, with both males and females exhibiting a mate preference for particular blends (*57*–*60*). In *D. melanogaster*, several hydrocarbon molecules have been identified as sex- and species-specific pheromones (*25*, *26*, *61*). For example, female dienes of 27 or 29 carbons in length (C27:2 and C29:2) are synthesized by a sex-specific oenocyte fatty acid elongase (EloF) and a desaturase (desatF) required for mating success (*47*–*49*). In summary, the available evolutionary evidence suggests that natural selection promotes long nonvolatile *n*-alkanes and methyl-branched alkenes for desiccation resistance, whereas sexual selection favors shorter semivolatile monoenes and dienes for pheromonal communication.

In contrast to the well-characterized sex-specific hydrocarbon barrier of the adult fly, little is known about how the pupa is protected from desiccation. Here, we use a combination of genetics, mass spectrometry, and a surface-specific analytical technique to identify the anatomical location and molecular composition of the desiccation barrier during early metamorphosis as well as its cellular origin and biosynthetic pathway.

## RESULTS

### Unisex hyperlong hydrocarbons coat the bodies of immature adults but not larvae or pupae

To characterize hydrocarbon profiles during *D. melanogaster* development, we optimized high-temperature gas chromatography mass spectrometry (HT-GC-MS) to resolve chain lengths of up to ∼40 carbons (C40; see Materials and Methods). Intriguingly, hexane extracts of the intact body surface (external face of the cuticle) analyzed using this method did not contain detectable amounts of hydrocarbons at the wandering third-instar larval (L3) or pupal (48 hours after pupariation) stages (Fig. 1A and table S1). In contrast, at 0 to 6 hours, after adult emergence (eclosion), young flies express abundant HCs on the external surface of their cuticle (Fig. 1A and table S1). The cuticular hydrocarbon profiles of 0- to 6-hour flies are remarkably similar in males and females, contrasting with marked sex-specific differences in older flies at 1, 3, and 7 days after eclosion (Fig. 1A, fig. S1, and table S1). Principal components analysis (PCA) indicated that the transition from unisex to male- and female-specific cuticular profiles is already evident at 1 day and very marked by 3 days (Fig. 1B). The range of cuticular hydrocarbon chain lengths switches from C27-C37 in young flies to C21-C31 in older flies, with an intermediate pattern at 1 to 3 days of age (Fig. 1C and fig. S1). Henceforth, we define C21 to C28 hydrocarbons as long (L) and C29 to C37 hydrocarbons as hyperlong (HL). The major categories of cuticular HL hydrocarbons are alkenes, dienes, and methyl-branched (me) alkanes, with much lower levels of linear alkanes. Although dienes are female restricted in mature adults, we observed HL dienes in both sexes at 0 to 6 hours after eclosion (Fig. 1A). During the first week after adult eclosion, both sexes transition to a lower cuticular ratio of HL to L hydrocarbons but females maintain a higher value, primarily because they continue to express C29-C31 hydrocarbons (Fig. 1, A and C). Together, the HT-GC-MS analyses reveal the unexpected absence of detectable hydrocarbons on the exterior body surface of larvae and pupae. They also show that hydrocarbons on the adult cuticle undergo a transition from unisex HL to sex-specific L profiles around the time of sexual maturation during the first day after eclosion.

**Fig. 1.**
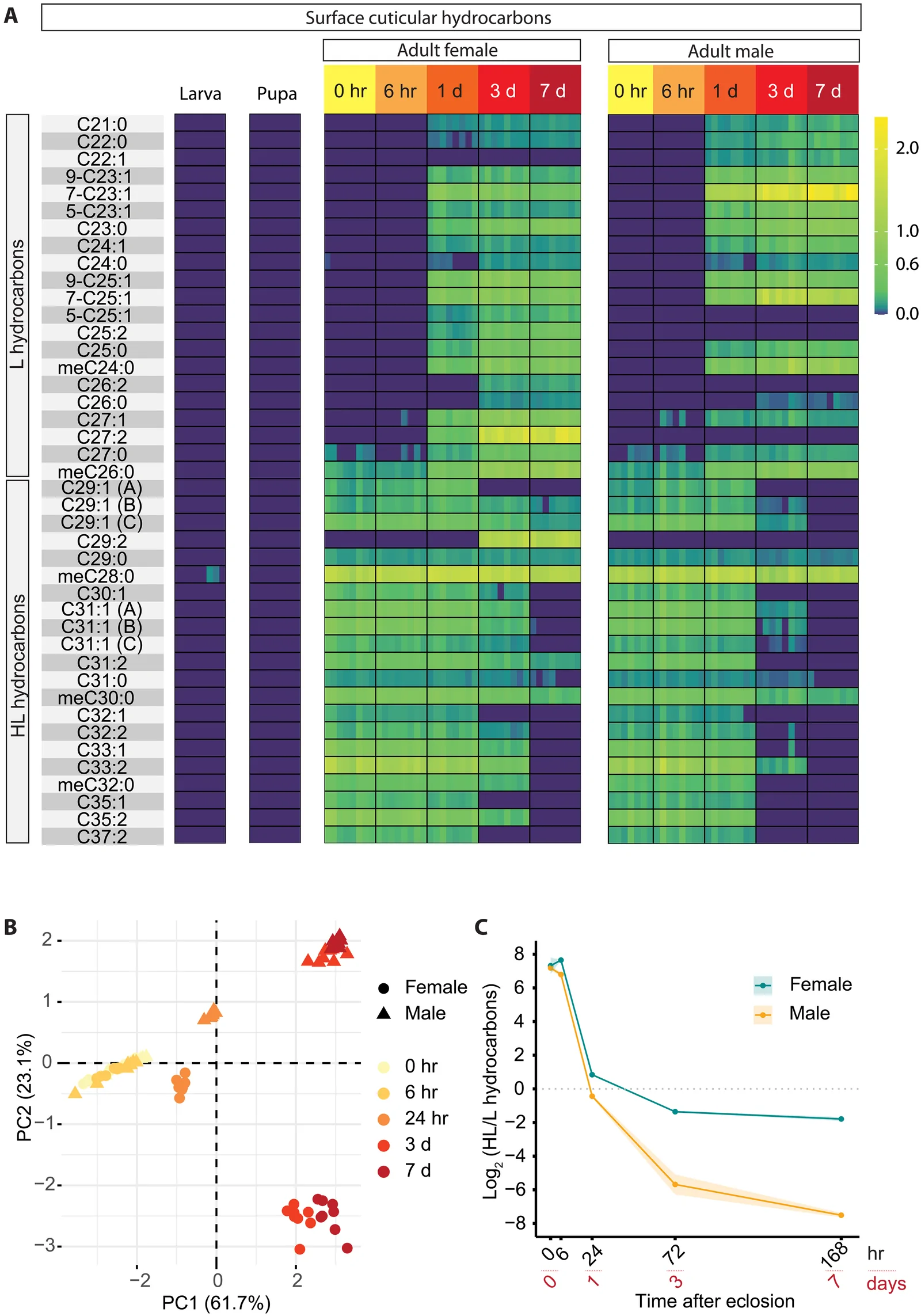
Cuticular hydrocarbons transition from unisex HL to shorter sex-specific profiles within a day of eclosion. (**A**) Heatmap of cuticular hydrocarbons from intact larvae (wandering L3 external larval cuticle), pupae (48 hours APF; external larval cuticle), and male and female adults (external adult cuticle at 0, 6, and 24 hours and 3 and 7 days after eclosion). A transition from unisex HL (HL-HCs) to long sex-specific (L-HCs) hydrocarbon profiles occurs between 6 and 24 hours after adult eclosion. Replicates are plotted next to each other within each box (data in table S1). hr, hours; d, days. (**B**) PCA of cuticular HCs from adult males (triangles) and females (circles) at 0 to 7 days of age. (**C**) Cuticular HL-to-L hydrocarbon ratios of male and female adults at 0 to 7 days after eclosion. Females maintain a subset of HL-HCs after sexual maturation. meC28 was excluded from the analysis due to its high abundance across all time points. In (B) and (C), the heatmap, PCA, and hydrocarbon ratios were determined from four replicates (*n* = 4) in each of two independent experiments (*N* = 2). In (C), mean values are plotted with shading indicating ±1 SD. Also see fig. S1.

### HL hydrocarbons are synthesized in larval oenocytes and stored in the fat body

It is unexpected that a hydrocarbon barrier is present on the exterior surface of the young adult but not the pupa, despite the pupa’s transition to a dry environment. This prompted us to investigate the developmental origin of HL hydrocarbons. Although wandering L3 larvae do not have detectable hydrocarbons on their external cuticle, following homogenization, substantial amounts of internal HL hydrocarbons and some L hydrocarbons (meC26 and C27:1) were observed (Fig. 2A). These internal hydrocarbons are similar, although not identical, in composition to those of newly eclosed adults, indicating that they may originate from a common source (Fig. 2A and table S2). Substantial amounts of all HL hydrocarbons are present in the larval fat body, whereas in the larval carcass (cuticle, epidermis, muscle, and larval oenocytes) and gut, only some methyl-branched hydrocarbons were detected (Fig. 2B and table S3). The levels of two major methyl-branched alkanes (meC28:0 and meC30:0) were nevertheless higher in the fat body than in the carcass or gut. The tissue-specific analysis indicates that HL hydrocarbons are stored internally during development, primarily within the fat body of the larva.

**Fig. 2.**
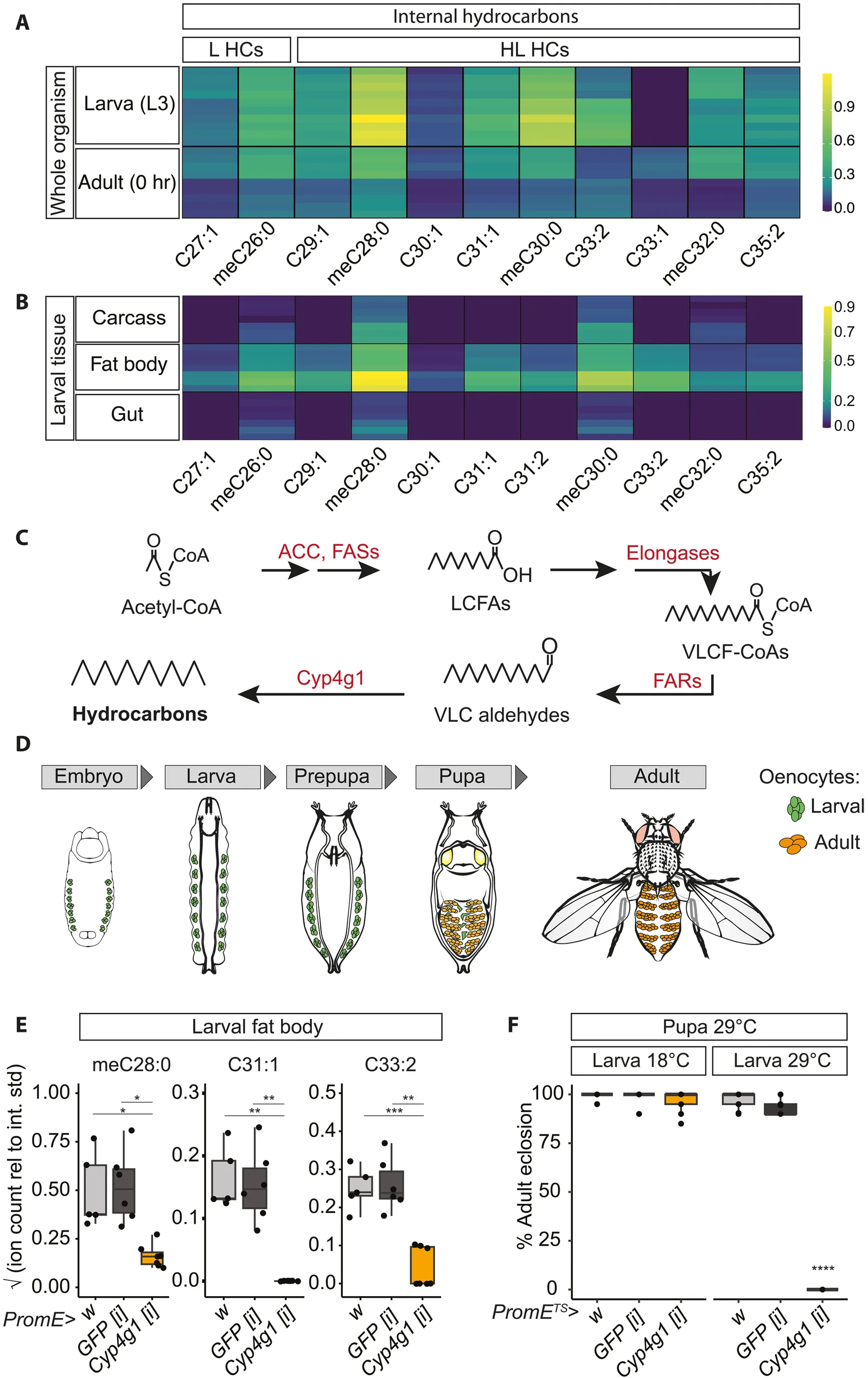
HL hydrocarbons are synthesized in larval oenocytes and stored in larval fat body. (**A**) Heatmap of internal hydrocarbons extracted from larvae (wandering L3) or newly eclosed adults (0 hours after eclosion). Both stages contain similar internal HL-HCs. *n* = 4, *N* = 2, and additional independent experiments are provided in table S2. (**B**) Heatmap of internal hydrocarbons extracted from the carcass, fat body, or gut of larvae (wandering L3). *n* = 3, *N* = 2, and additional independent experiments are provided in table S3. (**C**) Hydrocarbon biosynthetic pathway in *Drosophila*. Acetyl-CoA carboxylase (ACC) and fatty acid synthases (FASs) generate long-chain fatty acids (LCFAs) from acetyl-CoA. Fatty acid elongases then convert LCFAs to VLCF-CoAs. Fatty acid reductases (FARs) reduce VLCF-CoAs to very-long-chain fatty aldehydes (VLCF aldehydes), which are then converted to hydrocarbons via Cyp4g1, a P450 oxidative decarbonylase. (**D**) Schematic of separate larval (green) and adult (orange) oenocytes during *Drosophila* development. (**E**) *Cyp4g1* is required in larval oenocytes for HL-HC storage in the larval fat body. The *y* axis shows square root ion counts, relative to the internal standard, of an HL methyl-branched alkane (me-C28:0), an HL monoene (C33:1), and an HL diene (C33:2). The experimental and two control genotypes are, respectively, *PromE-GAL4* crossed to *UAS-Cyp4g1 RNAi* (*PromE>Cyp4g1[i]*) or to *UAS-GFP RNAi* (*PromE>GFP[i]*) or to *w[1118] iso31* (*PromE>w*). Statistical significance is indicated by *P < 0.05, **P < 0.005, and ***P < 0.0005. *n* = 2, *N* = 3. (**F**) *Cyp4g1S* is required in larval oenocytes for adult eclosion. *PromE^TS^* (*PromE-GAL4*, *UAS-GAL80^TS^*, and *UAS-CD8::GFP*), active at 29°C but not at 18°C, was crossed to *UAS-Cyp4g1 RNAi* (*PromE^TS^>Cyp4g1[i]*), *UAS-GFP RNAi* (*PromE^TS^>GFP[i]*), or *w[1118] iso31* (*PromE^TS^>w*). **** refers to *P* values lower than 0.00005. *n* = 3, *N* = 3.

We next determined the larval cell type that synthesizes HL hydrocarbons. In adults, sex-specific L hydrocarbons are known to be synthesized by adult oenocytes via multiple lipid metabolic enzymes including fatty acid elongases and the Cyp4g1 decarbonylase (Fig. 2C). However, adult oenocytes do not differentiate until late in pupal development and thus cannot be the site of synthesis for larval HL hydrocarbons. *D. melanogaster* and other dipterans have two separate generations of oenocytes, larval and adult, which differ from one another with respect to cell size, cell number, and developmental origin (Fig. 2D) (*39*, *62*–*67*). Larval oenocytes form in the embryo and play developmental roles in lipid metabolism, molting, and tracheal waterproofing (*43*, *68*). In contrast to their adult counterparts, larval oenocytes have not been implicated in the synthesis of cuticular hydrocarbons. Intriguingly, they are reported to express the hydrocarbon biosynthetic enzyme Cyp4g1 throughout embryonic and larval development (fig. S2A) (*43*, *69*). We therefore used an oenocyte-specific driver [*PromE-GAL4*; (*36*)] to express RNA interference (RNAi) for *Cyp4g1* throughout development. This knockdown resulted in viable larvae with markedly decreased fat body storage of methyl-branched alkane, monoene, and diene classes of HL hydrocarbons (represented by meC28:0, C31:1, and C33:2, respectively; Fig. 2E). Such larvae are able to pupariate but subsequently die during metamorphosis, before adult eclosion (fig. S2B) (*42*). *PromE-GAL4* was also combined with the temperature-sensitive Gal80 system (*70*) to knockdown *Cyp4g1* in oenocytes selectively during the pupal and/or larval stages. This revealed that oenocyte *Cyp4g1* is required during larval stages for subsequent adult eclosion and viability (Fig. 2F). The larval fat body expresses little or no *Cyp4g1* and its knockdown in this tissue with *Lpp-GAL4* is viable to adulthood (fig. S2, A and B). Together, the *Cyp4g1* and HT-GC-MS analyses indicate that larval oenocytes synthesize HL hydrocarbons, which are stored in the larval fat body and required for adult viability.

### EloHL is required in oenocytes for the biosynthesis of HL hydrocarbons

The carbon chain length of insect cuticular hydrocarbons is thought to be determined by the length of their very-long-chain fatty acyl-CoA ester (VLCF-CoA) precursors, which, in turn, depends on fatty acid elongases (Fig. 2C). To identify the elongase(s) responsible for synthesizing HL rather than L hydrocarbons, we purified actively translated mRNAs from larval versus adult oenocytes using a RiboTag method (*71*). Quantitative reverse transcription polymerase chain reaction (qRT-PCR) assays using RiboTag-purified mRNAs detected expression of 15 of the 20 predicted *D. melanogaster* elongases in the larval and/or adult oenocytes of males and/or females. PCA revealed that male and female elongase expression profiles cluster together in larval oenocytes (wandering L3 stage) but markedly separate in adult oenocytes (7 days after eclosion) (Fig. 3A). This correlates with the unisex versus sex-specific profiles of larval HL and adult L hydrocarbons, respectively. Principal component coefficients (loadings) of individual elongases show that the expression of two elongase genes, *CG6660* and *CG8534,* contributes most to larval versus adult oenocyte separation (Fig. 3B). These two elongase genes are highly expressed in the larval oenocytes of both sexes but *CG8534* is also highly expressed in the adult oenocytes of females but not males (Fig. 3C). We reasoned that the oenocyte-specific knockdown phenotype of an elongase dedicated to HL hydrocarbon biosynthesis would recapitulate or be less severe than that of *Cyp4g1*, which is larval viable but adult lethal (Figs. 2F and 3D). On the basis of this criterion alone, CG8534 appears to be a stronger candidate than CG6660 for an HL hydrocarbon elongase (Fig. 3D). Like *Cyp4g1*, *CG8534* is strongly expressed in the larval carcass (containing the oenocytes) but not in the larval fat body (fig. S2A). More direct evidence is that oenocyte-specific *CG8534* knockdown markedly decreases the internal HL hydrocarbons of larvae, relative to *GFP* knockdown and *GAL4* driver only controls (Fig. 3E). The decrease in HL hydrocarbons upon *CG8534* knockdown is accompanied by a corresponding increase in L hydrocarbons such as C27:1 and meC26:0 (Fig. 3E). This suggests that *CG8534* is specific for HL hydrocarbons, but to rule out that chain length selectivity arises from partial knockdown, or RNAi off-target effects, we used CRISPR-Cas9–induced homologous recombination (*72*) to generate a precise null allele, *CG8534*^Δ1^ (Fig. 3F and fig. S3B). *CG8534*^Δ1^ hemizygous [*CG8534*^Δ1^/*Df*(*3R*)*Excel6155*] larvae show a strong decrease in HL hydrocarbons and a concomitant increase in L hydrocarbons, similar to that seen with *CG8534* RNAi knockdowns (Fig. 3G). A time course of *CG8534*^Δ1^ hemizygotes indicates that HL hydrocarbon levels remain strongly decreased at 48 hours after puparium formation (APF) but have largely recovered by adult eclosion (fig. S3C and table S5). Thus, other elongases are likely to contribute to HL hydrocarbon synthesis during the pupal and adult stages. Both genetic analyses together demonstrate that *CG8534* is required in larval oenocytes for the synthesis of HL but not L hydrocarbons. They also suggest that loss of *EloHL* function diverts LCFA substrates toward other elongases that produce the shorter precursors used to make L hydrocarbons. On the basis of the selectivity of *CG8534* for HL hydrocarbons, we hereafter name it as *EloHL*.

**Fig. 3.**
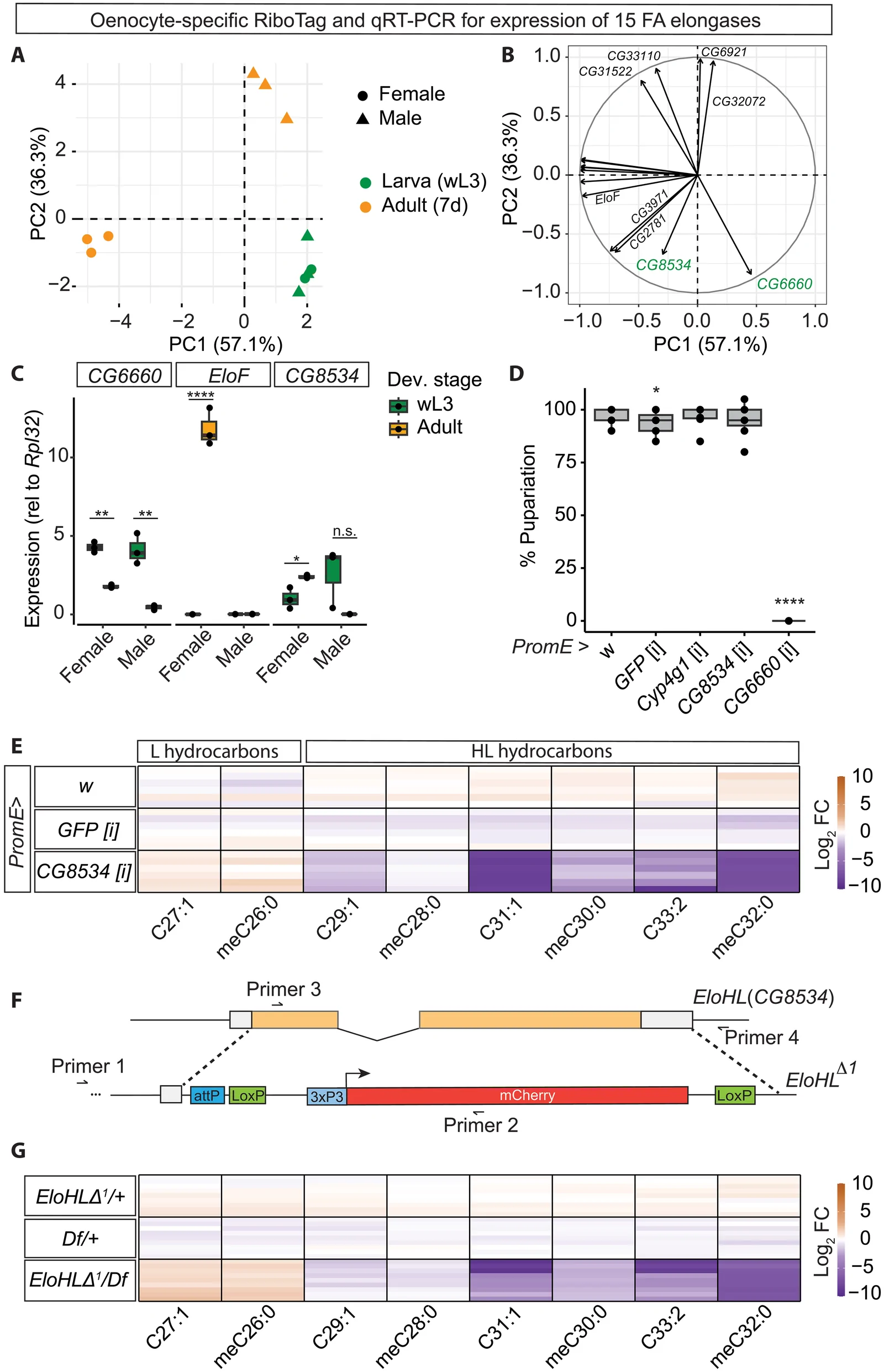
*EloHL* is required in larval oenocytes for the synthesis of HL hydrocarbons. (**A** and **B**) Oenocyte elongase expression transitions from unisex larval to sex-specific adult profiles. PCA (A) and loading plot (B) of the expression levels of 15 elongases detected in oenocyte RNA purified by RiboTag from male or female larvae (wandering L3) or adult flies (7 days after eclosion). *N* = 3. (**C**) Relative expression of *EloF*, *CG6660*, and *CG8534* (*EloHL*) in male and female oenocytes of larvae or 7-day adults. Statistical significance is indicated by *P < 0.05, **P < 0.005, and ****P < 0.00005; not significant (n.s.), *P* = 0.08. *N* = 3. (**D**) Viability of larvae with oenocyte-specific knockdown of *Cyp4g1*, *CG8534* (*EloHL*), or *CG6660* versus controls. Viability was measured by counting the number of puparia arising from a defined number of L1 larvae (% pupariation). Statistical significance is indicated by *P < 0.05 and ****P < 0.00005. *n* ≥ 3, *N* = 2. (**E**) CG8534 (*EloHL*) is required for HL-HCs. Heatmap shows log_2_ fold changes (log_2_ FC) of internal hydrocarbons of larvae (wandering L3) with oenocyte-specific *CG8534* knockdown (*PromE>CG8534[i]*), relative to the mean of both controls (*PromE>GFP[i]* and *PromE>w*). HL-HCs are decreased, whereas L-HCs (C27:1 and me-C26:0) are increased. Data in table S4. (**F**) Generation of *EloHL*^Δ^*^1^* null allele. The *EloHL* (*CG8534*) open reading frame (orange) and 3′UTR (gray) are replaced with a LoxP-flanked 3xP3-mCherry selection cassette and an attP site. Primers 1 to 4 were used to verify the resultant allele (fig. S3B). (**G**) *EloHL*^Δ^*^1^* mutant recapitulates HL hydrocarbon loss in oenocyte-specific *EloHL* knockdown. Heatmap shows internal HCs of hemizygotes [*EloHL*^Δ^*^1^*/*Df(3R)Excel6155*] or control heterozygotes [*EloHL*Δ*^1^*/*+* or *Df(3R)Excel6155*/*+*] at the wandering L3 larval stage. Log_2_ fold changes are indicated relative to the mean of both heterozygous controls. Data in table S4. Also see fig. S3.

### The developmental switch from HL to L hydrocarbons is conserved across *Drosophila*

To determine the prevalence of HL hydrocarbons across the *Drosophila* genus, we first examined a microsyntenic region that, in *D. melanogaster*, contains *EloHL* and four other predicted fatty acid elongase genes (*EloF*, *CG16904*, *CG9459*, and *CG9458*). Analysis of 30 species suggests that this microsynteny is restricted to *Sophophora* and not present in the *Drosophila* subgenus (Fig. 4A). Within the microsynteny of 23 *Sophophora* species, we found three examples of elongase duplications although many species lack a recognizable ortholog of *EloHL* and/or the other elongase genes, and three species contain only *CG16904* (Fig. 4A). HL hydrocarbons were detected in newly eclosed male and female flies of all six *Drosophila* species selected for HT-GC-MS analysis: two with a clear microsyntenic copy of *EloHL* (*D. melanogaster* and *D. biarmipes*) and four lacking it (*D. montana*, *D. ananassae*, *D. virilis*, and *D. mojavensis*) (Fig. 4B and fig. S4, A and B). Moreover, there is a clear unisex trend for hydrocarbon chain length shortening during the first week after eclosion in five of the six selected species (Fig. 4B and fig. S4, A and B). Nevertheless, the carbon chain length at the switch point is variable between the six species and, except for *D. biarmipes*, 7-day-old adults express C29 and some other hydrocarbons that we defined as HL in *D. melanogaster*. The desertic species *D. mojavensis* appears to be an exception to the hydrocarbon shortening trend as both males and females maintain very similar HL hydrocarbon profiles from eclosion to 1 week of age (Fig. 4B and fig. S4A). Together, these species comparisons show that a developmental switch to shorter hydrocarbons occurs across the *Drosophila* genus but it is more widespread than a recognizable microsyntenic ortholog of *EloHL*.

**Fig. 4.**
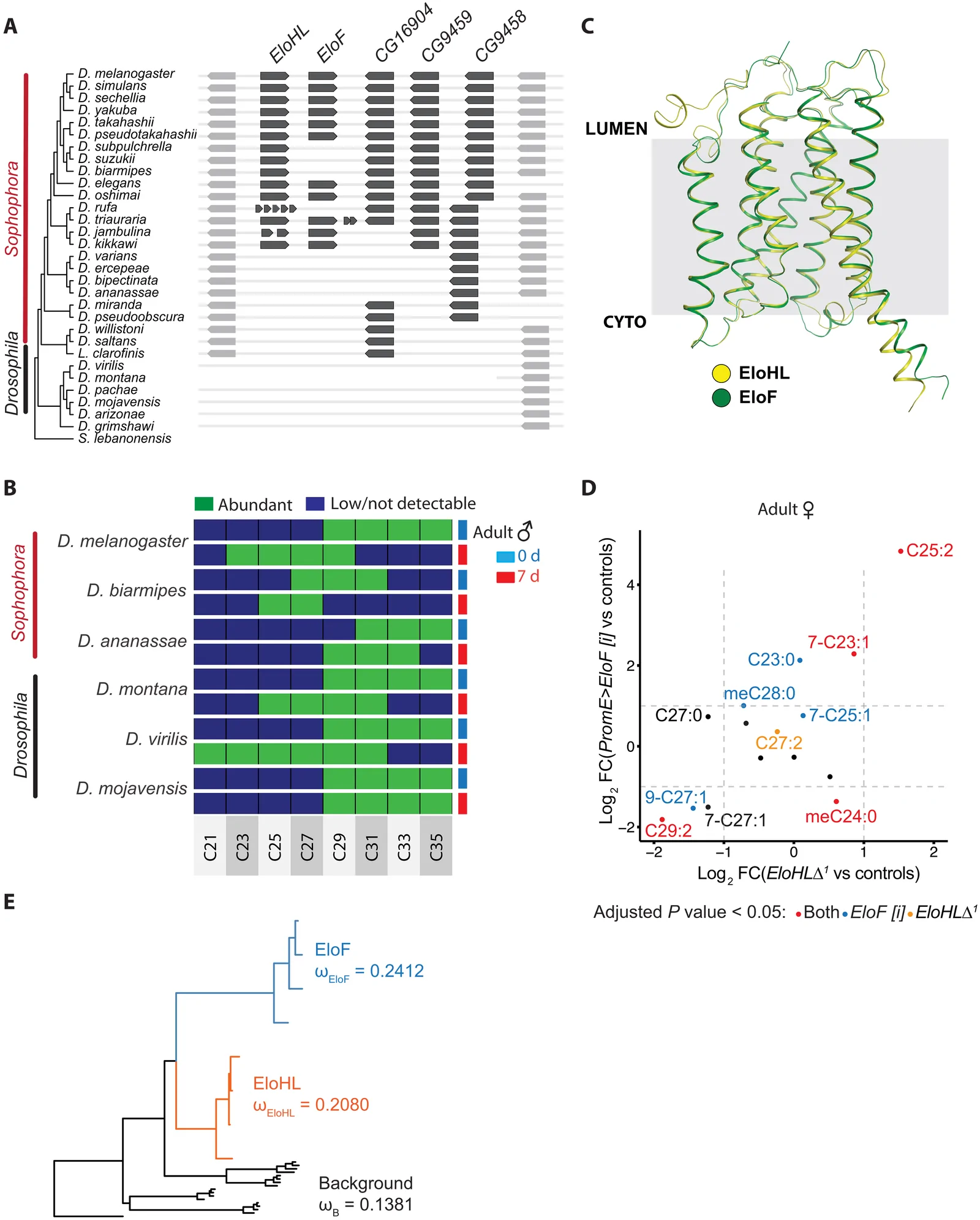
Hydrocarbon shortening in adult flies is widespread across the *Drosophila* genus. (**A**) Microsynteny of fatty acid elongases in the *EloHL* region across the *Sophophora* (including *D. melanogaster*) but not the *Drosophila* subgenus. Microsyntenies in *D. montana* and *S. lebanonensis* are partially identifiable or nonidentifiable, respectively. (**B**) Presence of cuticular hydrocarbons of immature (day 0) and mature (day 7) male flies from species in the *Sophophora* (*D. melanogaster*, *D. biarmipes*, and *D. ananassae*) and *Drosophila* (*D. montana*, *D. virilis*, and *D. mojavensis*) subgenera. Abundant (green) or low/not detected (blue) hydrocarbons are indicated according to length (C21 to C35) but pooling alkanes, methyl-branched alkanes, monoenes, and dienes. (**C**) Superposed AlphaFold-v4 structures of EloHL and EloF. Both structures are predicted to span the ER membrane from the cytoplasm (cyto) to the lumen. (**D**) *EloHL* and *EloF* are required for similar cuticular hydrocarbons in adult females (7 days). Correlation plot is shown of log_2_ fold changes for *EloF* (*y* axis) and *EloHL* (*x* axis). Colored dots indicate statistically significant (*P* < 0.05) changes in both *EloF* knockdown and *EloHL* mutants (red), *EloF* knockdowns only (blue), or *EloHL* mutants only (yellow). For *EloF*, adult oenocyte-specific knockdown used *PromE-GAL4*, *UAS-GAL80^TS^*, *UAS-CD8::GFP* (*PromE^TS^*>*EloF[i]*) and controls were *PromE^TS^>GFP[i]*. Hemizygotes [*EloHL*^Δ1^/*Df(3R) Excel6155*] were compared to both control heterozygotes [*EloHL*^Δ1^/*+* or *Df(3R)Excel6155*/*+*]. Significance was tested using analysis of variance (ANOVA) with Bonferroni correction. *n* = 3, *N* = 3. (**E**) *d*_N_/*d*_S_ (ω) ratios of fatty acid elongases in the *EloHL* microsynteny. ω for *EloF* but not *EloHL* is significantly higher (*P* = 0.005) than for the background elongase genes (*CG16904*, *CG9458*, and *CG9459*). Calculations are based on the elongase sequences from *D. melanogaster*, *D. simulans*, *D. sechellia*, and *D. yakuba*, each of which have five microsyntenic fatty acid elongases. The results of the different models tested are provided in table S6. Also see fig. S4.

Within the *EloHL* microsynteny, there is a related elongase gene (*EloF*) known to be required for the synthesis of hydrocarbon pheromones in adult females but not males (*47*, *49*). Hence, *EloF* is highly expressed in the adult oenocytes of females not males (Fig. 3C). There is, however, no detectable *EloF* expression in the larval oenocytes of either sex (Fig. 3C). *D. melanogaster* EloF and EloHL proteins are highly related (59% identity), and their AlphaFold predicted structures are closely superposed, suggesting that they could play similar roles (Fig. 4C). Consistent with this, a comparison of adult females 1 week after eclosion showed a strong correlation between the cuticular hydrocarbons that are up-regulated or down-regulated in *EloHL*^Δ^*^1^* mutants and in oenocyte-specific *EloF* knockdowns (Fig. 4D). Notably, loss of function of either elongase led to strong down-regulation of the female-specific pheromone C29:2 (nonacosadiene), whereas its shorter pheromonal counterpart, C27:2 (heptacosadiene), barely changed and C25:2 (pentacosadiene) was markedly up-regulated (Fig. 4D)*.* Some monoenes and methyl-branched alkanes were, however, differentially affected by the loss of function of each elongase. These findings strongly suggest that *EloHL* and *EloF* are required in the adult oenocytes of sexually mature females for the biosynthesis of overlapping sets of HL hydrocarbons.

To gain insight into the potential functional divergence of *EloHL* and *EloF* during evolution, we estimated selection pressures on coding sequences using phylogenetic analysis by maximum likelihood (PAML) (*73*). This method was used to calculate nonsynonymous/synonymous rate ratios (*d*_N_/*d*_S_ = ω) for four species (*D. melanogaster*, *D. simulans*, *D. sechellia*, and *D. yakuba*) with one copy of each of the five microsyntenic elongases. An additional gene, *CG31141*, outside the microsynteny, was also included due to its high sequence similarity to the other five elongases. For PAML analysis, four models (A to D) were generated (table S6). Model A assumes that all elongase clades have the same ω ratio. Models B and C propose respectively that the *EloF* or the *EloHL* clade has an independent ratio. Given that model B but not model C can reject model A (B versus A, *P* value = 0.00497; C versus A, *P* value = 0.121), it is likely that *EloF* has undergone substantially higher selection pressure than *EloHL*. Model D posits that both *EloHL* and *EloF* clades have independent ω ratios compared to the other “background” clades. This model outperforms the other three, significantly rejecting model A and model C (D versus A, *P* value = 0.00263; D versus C, *P* value = 0.00208) and marginally rejecting model B (D versus B, *P* value = 0.0457). Furthermore, model D gives the highest ω ratio for EloF (0.241), followed by EloHL (0.208) and then the other elongases (0.138) (Fig. 4E). Together, the phylogenetic models suggest that the coding sequence of *EloF* has undergone more functional changes than that of *EloHL* and the other elongases within the microsynteny.

### Larval synthesis of HL hydrocarbons protects the pupa from desiccation

*EloHL* could be more constrained than *EloF* during evolution because its larval oenocyte role in unisex HL hydrocarbon synthesis is essential for viability. Although *EloHL* is not required for the viability of larvae (Fig. 3, D to G), large amounts of HL hydrocarbons are stored in the larval fat body and could therefore be required later in development for an essential lipid barrier function. This prompted us to test whether *EloHL* might play a role in the pupal barrier to desiccation, which remains mysterious both in terms of its chemical composition and anatomical location. We first established that pupal dry weights of *EloHL*^Δ^*^1^*/*Df*(*3R*)*Excel6155* hemizygotes, deficient for HL hydrocarbons, as well as oenocyte-specific *Cyp4g1* knockdowns, lacking almost all hydrocarbons, are not very different from those of genetic controls (fig. S5, A and B). Pupal water content (wet minus dry weight) was then measured as a functional readout of the pupal lipid barrier, for individual animals subjected from 6 hours APF to varying degrees of desiccation stress induced by low relative humidity (RH) and/or high temperature. At 30% RH and 25°C, the pupal water content of genetic controls declined rapidly by 24 hours APF and subsequently slowed, such that it was roughly halved by 72 hours APF (fig. S5C). With this mild environmental stress, the temporal pattern and magnitude of pupal water loss in *EloHL*^Δ^*^1^* hemizygotes was very similar to that of genetic controls (fig. S5C). In contrast, with drier conditions of 15 to 20% RH, *EloHL*^Δ^*^1^* hemizygotes lost more water by 48 hours APF than controls, and this was observed across temperatures from 25° to 28°C (Fig. 5A). At 29°C, these drier conditions produced catastrophic loss of ∼75% of pupal water by 48 hours APF, both in hemizygotes and in genetic controls (Fig. 5A). At 15 to 20% RH and 26°C, the higher dehydration rate of *EloHL*^Δ^*^1^* hemizygotes was already apparent by 24 hours and continued until 48 hours APF (Fig. 5B). The dehydration assays demonstrate that *EloHL* is required for an efficient pupal desiccation barrier. Together with the previous HT-GC-MS analysis, these data suggest that the desiccation barrier involves HL hydrocarbons.

**Fig. 5.**
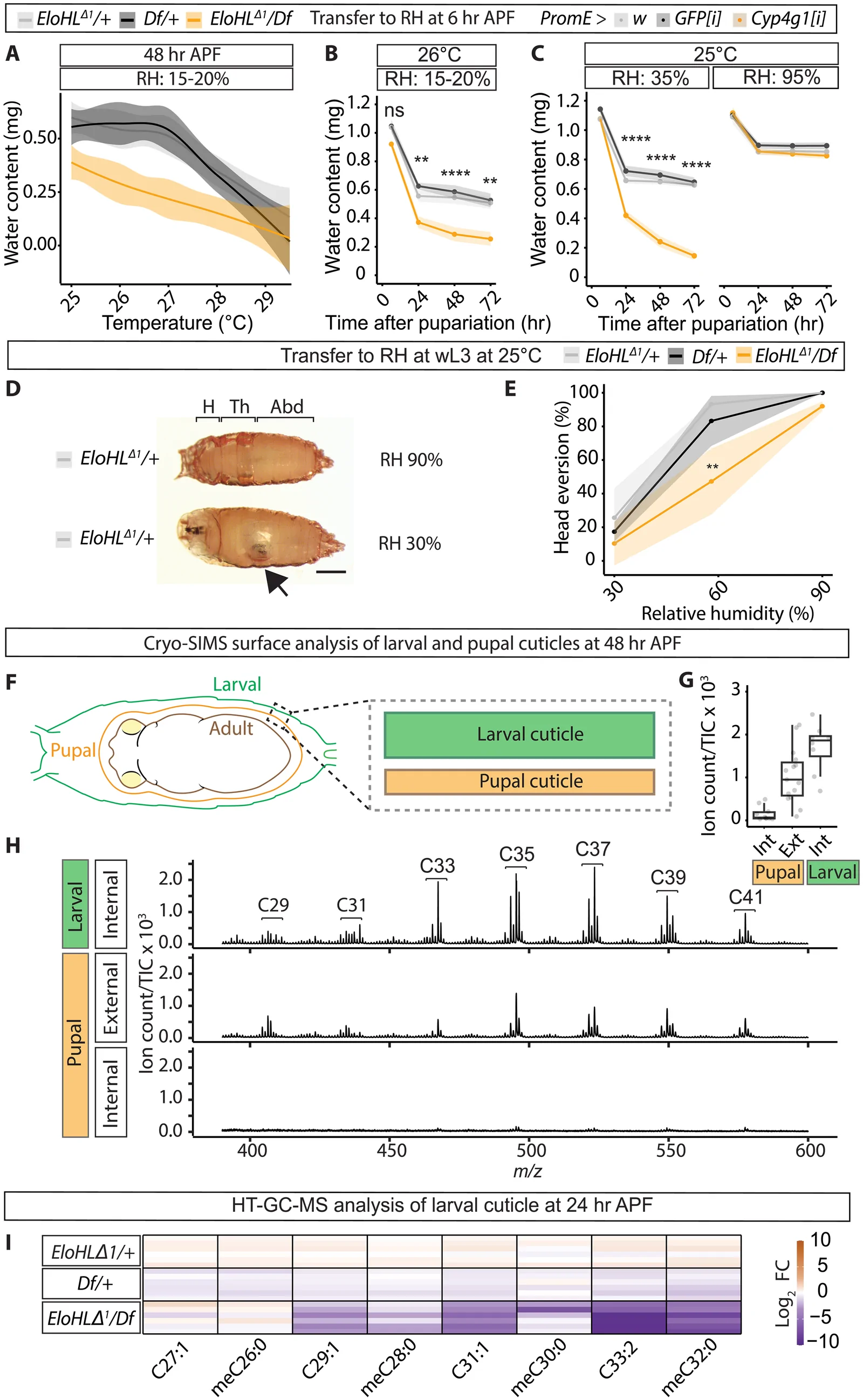
*EloHL*-dependent HL hydrocarbons on the internal larval cuticle protect the prepupa from desiccation. (**A**) Pupal water content versus temperature for *EloHL*^Δ1^/*Df*(*3R*)*Excel6155* (*EloHL*^Δ1^) hemizygotes and heterozygous controls (*EloHL*^Δ1^*/+* or *Df*(*3R*)*Excel6155/+*) at 48 hours APF with 15 to 20% RH. *EloHL*^Δ1^/Df increases sensitivity to water loss at lower temperatures. *n* ≥ 5, *N* = 6. (**B**) Pupal water content (mg) of *EloHL*^Δ1^ hemizygotes and heterozygous controls with 15 to 20% RH at 26°C. *n* ≥ 5, *N* = 4. (**C**) Time course of the pupal water content of oenocyte-specific *Cyp4g1* knockdown and control animals at 25°C, with 35 or 95% RH. Oenocyte-specific knockdown of *Cyp4g1* leads to excess water loss at 35% RH but not 95% RH. *N* = 2, *n* = 10. (**D**) Morphology of *EloHL*^Δ1^/*+* heterozygotes at 24 hours APF, with 30 or 90% RH at 25°C. At 30% RH, abnormal persistence of the internal gas bubble (arrow) and no distinct head (H), thorax (Th), and abdomen (Abd) indicate failed head eversion. Scale bar, 500 μm. (**E**) Head eversion (%) in *EloHL*^Δ^*^1^* hemizygotes or control heterozygotes. *n* = 1, *N* = 4. Animals were transferred to the indicated RH at 6 hours APF (A to C) or wL3 (D and E). (**F**) Diagram of the three cuticles of the *Drosophila* pupa, external to internal: larval (puparium), pupal, and adult. (**G**) Cryo-ToF-SIMS hydrocarbon signal quantification (C29-C37) of the pupal internal, pupal external, and larval internal cuticles at 48 hours APF. *n* ≥ 5, *N* = 2. (**H**) Corresponding cryo-ToF-SIMS spectra from (G) showing HL hydrocarbons from C29 to C41 in length. (**I**) HT-GC-MS heatmap of HL hydrocarbons from dissected larval cuticles at 24 hours APF. HL hydrocarbons are strongly depleted in *EloHL*^Δ^*^1^* hemizygotes compared to both control heterozygotes. Log_2_ fold changes relative to the mean of both heterozygous controls. Data in table S7. *n* = 2 to 4, *N* = 2. Statistical significance is indicated by **P < 0.005 and ****P < 0.00005.

*EloHL* mutants and larval oenocyte-specific *EloHL* knockdowns both retain L hydrocarbons, such as C26 and C27 molecules, during development. This contrasts with the almost complete lack of all hydrocarbons in *Cyp4g1* knockdowns (Fig. 2E). With mild desiccation stress (30% RH and 25°C) that does not elevate dehydration in *EloHL* mutants, water loss in larval oenocyte-specific *Cyp4g1* knockdown pupae is nevertheless very pronounced, almost double that of genetic controls (Fig. 5C). Excess water is already lost at 24 hours APF, 3 days before eclosion and long before adult oenocytes differentiate and synthesize hydrocarbons (Fig. 5C). Excessive water loss in *Cyp4g1* knockdown pupae at 25°C could be fully rescued by raising the humidity from 30 to 95% RH (Fig. 5C). The stronger pupal dehydration phenotype of *Cyp4g1* compared to *EloHL*, together with the HT-GC-MS analysis, strongly suggests that L and HL hydrocarbons both contribute to the pupal desiccation barrier. Nevertheless, loss of only the HL class in *EloHL* mutants is enough to compromise barrier efficiency in high-temperature and/or arid environments. To determine how the compromised desiccation barrier in *EloHL* mutants affects the progression of metamorphosis, we quantified head eversion. This process is essential for adult eclosion and specifically marks the developmental transition from prepupa to pupa, which occurs at ∼12 hours APF at 25°C (*74*, *75*). We found that most *EloHL*^Δ^*^1^* or *Df*(*3R*)*Excel6155* heterozygous controls completed head eversion when exposed to 60 or 90% RH (although not 30% RH) from the wandering L3 larval stage onward (Fig. 5, D and E). More than half of *EloHL*^Δ^*^1^* hemizygotes, however, fail to complete head eversion at 60% RH (Fig. 5E). These results, together with the water loss assays, indicate that the *EloHL*-dependent desiccation barrier is required for the prepupal-to-pupal transition in challenging arid environments.

The results thus far raise important questions about the anatomical location of the *EloHL*-dependent hydrocarbon barrier to desiccation. Multiple cuticles are synthesized during insect development, and in holometabolous insects like *Drosophila*, three of these are present during some or all of metamorphosis. From external to internal, these are the third-instar larval cuticle, which hardens to form the puparium (external pupal case), the thinner and more flexible pupal cuticle surrounding the developing adult, and the newly formed adult cuticle itself (Fig. 5F). The puparium, henceforth called the larval cuticle, persists throughout metamorphosis and the pupal and adult cuticles are formed at ∼12 and ∼36 hours APF, respectively (*76*, *77*). Hence, compromised larval and/or pupal but not adult cuticular barriers could, in principle, contribute to the excessive water loss observed at 24 hours APF in *EloHL* mutants and *Cyp4g1* knockdowns. To investigate this further, we analyzed dissected cuticles from pupae at 48 hours APF using a surface-specific analytical technique called cryogenic secondary ion mass spectrometry (cryo-SIMS) that we had previously optimized for adult cuticular hydrocarbons (*78*). The wild-type larval cuticle lacks hydrocarbons on its external surface (Fig. 1A) but signals for HL hydrocarbons ranging from C29 to C41 are very abundant on its internal surface (Fig. 5, F to H). For the pupal cuticle, somewhat less abundant HL hydrocarbon signals are also present on the external but not the internal surface (Fig. 5, F to H). Similar HL hydrocarbon signals are also present on the external pupal cuticles of *D. mojavensis* and *D. virilis*, suggesting that this may be widespread across the *Drosophila* genus (fig. S6). We then analyzed the external pupal cuticle of *EloHL*^Δ^*^1^* hemizygotes, but HL hydrocarbon signals were at least as abundant as in the control genotypes at 48 hours APF (fig. S5F). Notably, however, larval cuticles dissected as early as 24 hours APF and analyzed at high chemical resolution via HT-GC-MS showed that HL hydrocarbons are very strongly depleted from this lipid barrier in *EloHL*^Δ^*^1^* hemizygotes (Fig. 5I). Together with the dehydration and head eversion assays, this key finding strongly suggests that an HL hydrocarbon barrier to desiccation, located on the internal face of the larval cuticle, is essential for the prepupal-to-pupal transition during metamorphosis in an arid environment.

In summary, the results of this study identify a unisex HL hydrocarbon barrier that is synthesized by *EloHL* in larval oenocytes, stored in the larval fat body, and then mobilized to the internal surface of the larval cuticle to protect early metamorphosis from desiccation.

## DISCUSSION

This *Drosophila* study fills a gap in our understanding of how insects cope with the threat of dehydration during metamorphosis. It identifies the cell source, chemical basis, synthetic mechanism, and anatomical location of a lipid barrier specialized for the pupa, a developmental stage vulnerable to water loss. The pupal lipid barrier differs from its adult counterpart in four important ways. First, it is synthesized by larval oenocytes, a separate cell population from the adult oenocytes that produce the lipid barrier of the imago. Second, it is stored during larval development in the fat body until required at the pupal stage. Third, it is composed of unisex hydrocarbons with longer carbon chain lengths, on average, than those of the sex-specific adult hydrocarbons. Fourth, its anatomical location corresponds to the larval and the pupal cuticles, structures distinct from the adult cuticle. We now discuss how distinct hydrocarbon barriers are synthesized during metamorphosis and adulthood and how their evolution may be shaped by different selective pressures.

### Larval and adult oenocytes synthesize distinct hydrocarbon barriers

Despite their shared name, larval and adult oenocytes are different cells with distinct developmental origins and functions (*37*, *38*, *43*, *63*, *68*, *79*). Our study nevertheless demonstrates that both cell types share an overlapping function in the *Cyp4g1*-dependent synthesis of hydrocarbon lipid barriers, albeit chemically distinct ones. Larval oenocytes synthesize HL hydrocarbons of the pupal and immature adult cuticle, whereas their adult namesakes make sex-specific shorter hydrocarbons of the mature adult cuticle. In contrast to a previous interpretation (*42*), we find that the larval not the adult oenocytes make the hydrocarbons relevant for survival at adult eclosion. The different carbon chain lengths that each type of oenocyte produces appear to be specified by distinct fatty acid elongases. One of these, EloHL, is required in larval oenocytes to produce the unisex HL hydrocarbons that coat the internal surface of the hardened larval cuticle and contribute to the desiccation barrier during early metamorphosis. Given that *EloHL* mutants have a weaker desiccation phenotype than *Cyp4g1* knockdowns and that they retain HL hydrocarbons on the pupal cuticle, it is likely that other fatty acid elongases contribute to the overall pupal barrier to water loss. Genes encoding candidate elongases expressed in the larval oenocytes of both sexes include *CG6660*, which is known to be required for larval tracheal waterproofing and viability (*68*). Adult oenocytes on the other hand express a range of sex-biased elongases, including female-specific EloF, which produces C29 and shorter hydrocarbons with dual barrier and pheromonal functions. Our results and those of others (*80*, *81*) together indicate that *EloHL* is not only expressed in a unisex pattern in larval oenocytes but also redeployed later in a sex-specific manner in adult oenocytes to synthesize female pheromones such as 7,11-nonacosadiene (C29:2).

A key finding of this study is that larval oenocytes produce HL hydrocarbons but store them in the fat body rather than transporting them immediately to the surface of the larval cuticle. Larval oenocytes undergo cell death at the midpupal stage (*43*) and thus cannot supply hydrocarbons to the cuticle during late pupal stages. The persistence of larval fat body cells throughout metamorphosis (*82*, *83*), however, enables it to bridge the developmental gap between the larval and adult oenocyte sources of hydrocarbons (Fig. 6). The mechanisms of hydrocarbon transport between oenocytes, fat body, and cuticles during metamorphosis are currently unknown but could involve lipoproteins as has been shown in other insects (*84*, *85*).

**Fig. 6.**
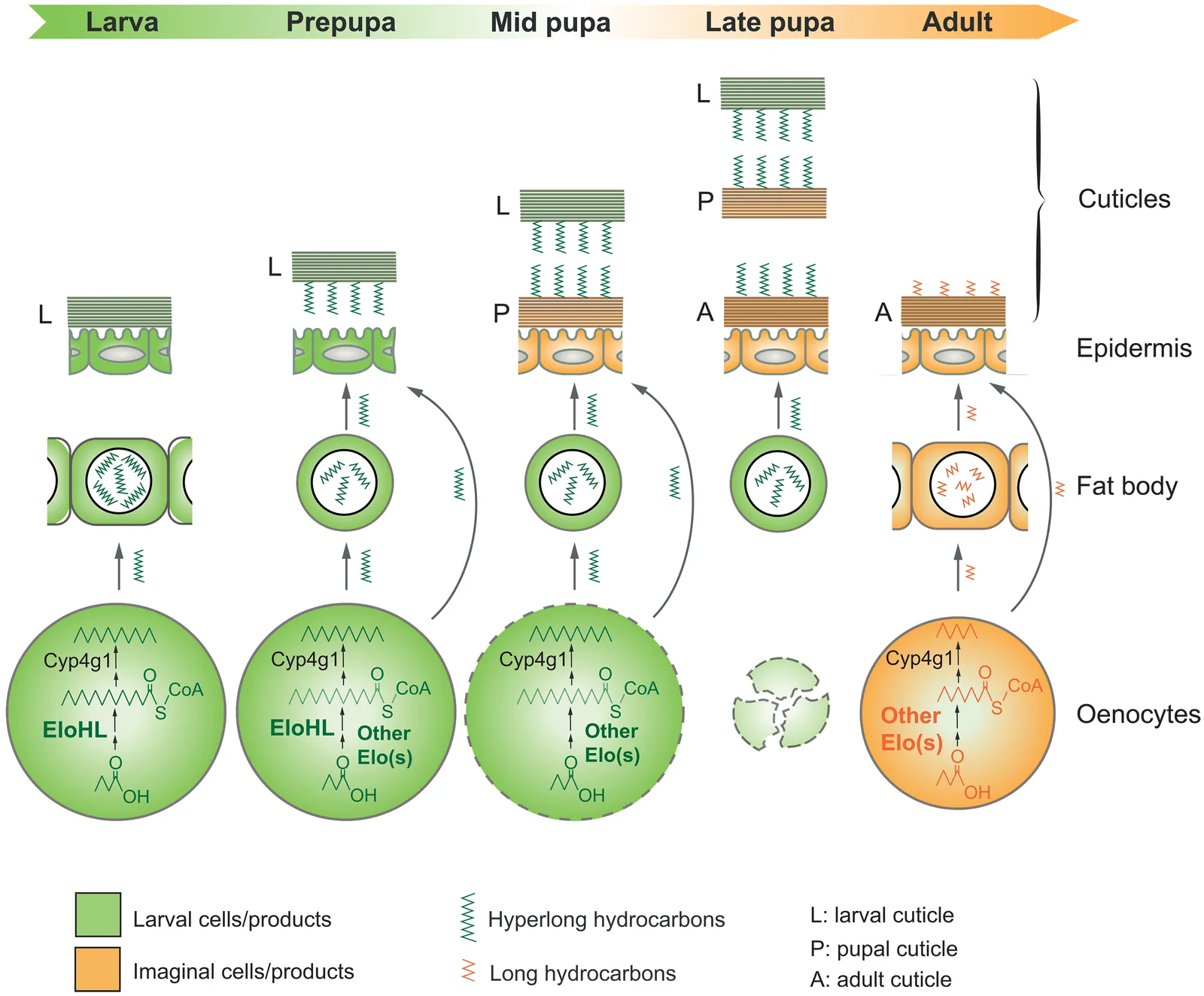
Model for biogenesis of distinct larval, pupal, and adult cuticular lipid barriers. *Drosophila* develop via larval, pupal, and adult stages, each of which secretes a distinct cuticle. Larval epidermal cells (green) secrete the larval cuticle (L), whereas imaginal epidermal cells (orange) secrete the adult cuticle (A). Both larval and imaginal epidermal cells are thought to secrete the pupal cuticle (P) (*102*), but, for clarity, only the imaginal contribution is shown. In the larva, unisex HL (C29 and longer) hydrocarbons are synthesized in larval oenocytes via the fatty acid elongase EloHL and the cytochrome P450 enzyme Cyp4g1. At later stages, both larval and adult oenocytes may also use other elongases including *CG6660*. In the larva, HL hydrocarbons are stored within the larval fat body, a tissue retained throughout metamorphosis. After pupariation, the desiccation barrier of the prepupa requires HL hydrocarbons to be mobilized (arrows) from the larval fat body and perhaps also directly from the larval oenocytes—initially to the internal surface of the larval cuticle (puparium) and, after the prepupal-to-pupal transition, to the external surface of the pupal cuticle. In the mid pupa, the adult cuticle has formed and larval oenocytes have largely undergone programmed cell death (*43*). At this stage, HL hydrocarbons are transported from larval fat body cells to the adult cuticle. In the adult, a separate population of adult oenocytes synthesizes long hydrocarbons (C28 and shorter) that replace HL hydrocarbons and play dual desiccation barrier and pheromonal roles. In mature females, adult oenocytes use EloHL and other elongases such as EloF to synthesize some HL hydrocarbons, such as C29 pheromones. The storage and transport mechanisms for *Drosophila* cuticular hydrocarbons have yet to be characterized in detail.

### Specialized lipid barriers for distinct life stages and ecological niches

We found that the larva, which lives in a semiaquatic environment, lacks detectable hydrocarbons on its cuticular surface. In contrast, the pupa occupies a drier terrestrial niche and is coated with a unisex profile of HL hydrocarbons. Physicochemical and biological studies of insect hydrocarbons indicate that longer chain lengths and higher proportions of methyl-branched and linear alkanes promote lower volatility and higher desiccation resistance (*5*, *29*, *53*). HL hydrocarbons of the pupal lipid barrier are therefore likely to have evolved to minimize water loss but presumably they are not volatile enough to function well as olfactory pheromones, at least not in a temperate climate. For this reason, adult flies of those *Drosophila* species that communicate using sex-specific olfactory pheromones may need to transition to shorter more volatile hydrocarbons. These shorter hydrocarbons appear to occupy a physicochemical sweet spot, long enough to support barrier function yet short enough for some of the monoenes and dienes to work as volatile olfactory pheromones.

Our evolutionary survey, together with previous studies (*50*, *86*, *87*), indicates that the transition to shorter hydrocarbons during adult sexual maturation is widespread across the *Drosophila* genus. We found, however, that HL hydrocarbons persist in 7-day adults of the desert species *D. mojavensis* and suggest that this may reflect strong ecological pressure for desiccation resistance in an arid environment. *D. mojavensis*, like some other species expressing HL hydrocarbons, does not have a recognizable microsyntenic ortholog of *EloHL*. In these species, *EloHL*-related elongases elsewhere in the genome may synthesize HL hydrocarbons. In addition, it was recently shown that an elongase (mElo) in *D. mojavensis* can elongate methyl-branched hydrocarbons up to 33 carbons in length and these branched molecules are thought to contribute to desiccation resistance in arid environments (*45*, *53*). The low volatility of HL and methyl-branched hydrocarbons makes them both well suited as barrier lipids but not as volatile pheromones, perhaps explaining why some *D. mojavensis* subspecies may rely more during courtship on nonolfactory cues (*88*).

EloHL contributes to both desiccation resistance and female pheromone biosynthesis, overlapping in the latter function with EloF. Our sequence analysis suggest that EloHL has likely evolved with greater negative purifying selection than EloF. We speculate that this pattern might be explained by a constrained unisex function of EloHL in the pupal desiccation barrier versus a shared more rapidly evolving function in adult sex pheromones (*17*, *25*, *36*, *47*, *55*, *89*). More generally, the findings in this study highlight that the pupal desiccation barrier of *D. melanogaster* is not able to protect metamorphosis from lethal desiccation when low humidities and high temperatures are combined. These humidity and temperature values are found in many areas of our planet, so given the pace of climate change, *D. melanogaster* and some other species may struggle to adapt rapidly enough to survive within some or all of their current geographical ranges.

## MATERIALS AND METHODS

### Drosophila stocks

*D. melanogaster* was maintained on yeast-glucose-cornmeal-agar food (*90*) at 25°C and 60% RH with a 12-hour light/12-hour dark cycle unless otherwise specified. Other *Drosophila* species were maintained on yeast-glucose-cornmeal-agar medium with the addition to the surface of ∼3 ml of *Drosophila* Quick Mix Medium (Blades Biological Ltd., #DTS070), at room temperature (∼25°C, ∼40% RH). The *D. melanogaster* GAL4 driver lines used in this study were as follows: *PromE* (*w*; *PromE-GAL4*, *UAS-CD8::GFP*/*CyO*), *PromE^TS^* (*w*; *PromE-GAL4*, *UAS-CD8::GFP*, *UAS-tub-GAL80^TS^*/*CyO*) (*36*), and *Lpp* (*Lpp-GAL4/TM6b*, *Tb*, *Dfd-YFP*) (*91*). *UAS-RNAi* lines were obtained from the Vienna Drosophila RNAi Stock Centre (VDRC) or the Bloomington Drosophila Stock Centre (BDSC) [*CG8534*: 106515 (VDRC); *CG6660*: 62422 (BDSC); *EloF*: 44660 (BDSC); *Cyp4g1*: *UAS-Cyp4g1.WIZ* (see below)]. We also tested another RNAi line for *CG8534* (BDSC #53299), but this gives an off-target eclosion defect and was therefore excluded. Genetic crosses were set up in small cages and eggs/larvae were collected from grape juice agar plates [25% (v/v) grape juice, 1.25% (w/v) sucrose, and 2.5% (w/v) agar] and transferred to vials containing yeast-glucose-cornmeal-agar food. *Drosophila* species originated from the National *Drosophila* Species Stock Centre (NDSSC) with the following stock numbers: *D. biarmipes* (14023-0361.10), *D. ananassae* (14024-0371.30), *D. montana* (15010-1021.24), *D. virilis* (15010-1051.118), and *D. mojavensis* (15081-1352.22).

For stage-specific knockdown of *Cyp4g1* in oenocytes, the temperature-sensitive *PromE^TS^* driver was used. Larvae were maintained at either 18° or 29°C during development, and larvae raised at 18°C were transferred to 29°C when pupariation occurred. Pupariation and adult eclosion were quantified from animals raised in vials containing yeast-glucose-cornmeal-agar food inside a temperature- and humidity-controlled room or incubator. RH values in the figure legends refer to the ambient humidity. The RH inside fly vials containing yeast-glucose-cornmeal-agar food was measured at ∼90%. Pupariation was quantified by counting the number of pupae formed 6 days after transferring 20 L1 larvae of the appropriate genotype to a yeast-glucose-cornmeal-agar food vial. Adult eclosion was quantified by counting the number of adults that successfully emerged from the pupal case.

### Generation of *EloHL*^Δ^*^1^* null allele and *UAS-Cyp4g1* RNAi

The *EloHL*^Δ^*^1^* mutant was generated by CRISPR-Cas9–induced homologous recombination using the TVΔattp-Pax-mCherry and CFD4 vectors (*72*, *92*). The TVΔattp-Pax-mCherry vector features an attP site and an excisable 3xP3-mCherry cassette flanked by LoxP sites, enabling tracking of the mutation via fluorescent red eyes. Homology arms flanking the deletion breakpoints at the ends of the 5′ and 3′ untranslated region (UTRs) of *CG8534* were cloned into the TVΔattp-Pax-mCherry vector using the following primers: Homology_1_fw, Homology_1_rv, Homology_2_fw, and Homology_2_rv. Genomic deletions were achieved by coinjecting TVΔattp-Pax-mCherry with the CFD4 vector, which expresses two guide RNAs targeting the deletion breakpoints at the ends of the 5′UTR (CFD4_T1_CG8534) and 3′UTR (CFD4_T2_CG8534) of *EloHL/CG8534*. The vectors were coinjected into the *nos-Cas9-mSA* fly line with an attP2 landing site, which carries *Pax-GFP* as a genetic marker. Integration of the TVΔattp-Pax-mCherry cassette was confirmed by PCR analysis using Primers 1 and 2. Primer 1 is located outside the homology arm used to integrate the cassette, whereas Primer 2 is located within the mCherry sequence. In addition, the absence of *CG8534* was confirmed in hemizygous mutants [*EloHL*^Δ^*^1^/Df(3R)Excel6155*] using PCR with Primers 3 and 4, which amplify part of the *CG8534* cDNA. All primer and guide RNA sequences are listed in table S8.

To generate *Cyp4g1* RNAi lines, the cDNA clone GH05567 was used as a template to amplify a 330–base pair (bp) *Cyp4g1* fragment with Pfu polymerase using the 5′-tagged primers Cyp4g1_fw and Cyp4g1_rv (table S8). The resultant PCR product was cloned into the UAS-RNAi vector pWIZ, which produces intron-spliced hairpin RNA (*93*). The final Cyp4g1.WIZ construct was integrated into the genome of a *y^1^ w^1118^* strain by P-element-mediated transformation using standard methods (*94*).

### Sample preparation for HT-GC-MS

For analysis of surface hydrocarbons, five larvae or pupae were collected from the side of the vial with a paintbrush, washed with phosphate-buffered saline (PBS), dried on tissue paper, and weighed in analysis groups on a microbalance (Sartorius Lab Instruments, Sartorius Cubis MSE3.6P-000-DM). Adults were anesthetized with CO_2_ and weighed on a microscale. Pooled individuals were washed in 50 μl of hexane with 0.1 mM octadecane (internal standard) in a GC vial (Agilent Technologies, #5182-0715) for 2 min by agitating the solution. The hexane extract was then transferred to a new GC vial and capped. For analysis of internal hydrocarbons, five (or 10 for the data in fig. S3C) larvae, pupae, or adults were first processed as above to remove external surface hydrocarbons. For analysis of specific internal tissues, fat body, inverted carcass (with all internal tissues and the head removed), and the remaining internal organs (gut, Malpighian tubules, and crop) referred to as gut were dissected from 10 larvae in PBS. Larval cuticles (puparia) were dissected from ∼30 individuals after head eversion, at ∼24 hours APF. Pooled tissues or whole animals were transferred to a metal Precellys tube with 500 μl of hexane with 0.01 mM octadecane (internal standard) and homogenized using the Precellys Evolution tissue homogenizer (Bertin Technologies). Samples were homogenized for four rounds of 25 s at 6000 rpm, transferred to a 1.5-ml microcentrifuge tube, and centrifuged for 10 min at 14,000 rpm. The supernatant was then transferred to a GC vial, and the hexane was allowed to evaporate overnight in a fume hood. Samples were then reconstituted in 50 μl of hexane, and the GC vials were capped.

### High-temperature gas chromatography–mass spectrometry

HT-GC-MS used an Agilent 7890B-7000C GC-MS system in EI mode, with the exception of the data in Fig. 5I, which used an Agilent 7890B-5977A GC-MS system in EI mode. Data acquisition and analysis were performed using MassHunter (version 10.0, Agilent Technologies Inc.). Splitless injection of 1 μl (injection temperature: 310°C) onto a BPC5 column (30 m length, 0.25 mm width, 0.25 µm film thickness, plus 10 m guard column, SGE), with helium as the carrier gas. The initial oven temperature was 100°C (1 min), followed by a temperature gradient to 350°C at 10°C/min with an optional hold time of 25 min used for internal and whole animal hydrocarbon samples. Sample running order was randomized using the MassHunter sample sequence randomizer. Data analysis was performed using the MassHunter Qualitative and Quantitative Analysis (version 10.0, Agilent Technologies). Peak areas were first normalized to an internal standard (octadecane) spiked into the sample at the time of metabolite extraction. Compounds were identified by referencing commercial standards when available or by retention time and fragmentation pattern when standards were unavailable. After normalization to the internal standard, data were square root transformed for visualization.

### RiboTag RNA isolation and qRT-PCR

The oenocyte-specific RiboTag protocol was adapted from a previous study (*71*). *PromE-Gal4* driver virgins were crossed to male *UAS-RPL13a-Flag* flies (BDSC #83684). Eggs were collected from grape juice agar plates and transferred to fly vials with yeast-glucose-cornmeal-agar food. Thirty male or female wandering L3 larvae or adult flies were snap frozen in liquid nitrogen and stored at −80°C. On the day of RiboTag analysis, samples were thawed on ice and transferred to plastic Precellys tubes filled with ice-cold lysis buffer [20 mM Hepes-KOH (pH 7.4), 12 mM MgCl_2_, 100 mM KCl, 1% NP-40, 1 mM dithiothreitol (DTT), cycloheximide (100 μg/ml), and RNAsin protease inhibitor (40 U/ml)]. The samples were homogenized for two rounds (25 s at 6000 rpm) using the Precellys Evolution tissue homogenizer (Bertin Technologies). The lysate was transferred to a new tube and centrifuged for 10 min at 10,000 rpm at 4°C. Sample lysate was precleared by adding it to Protein G Dynabeads, previously washed three times with wash buffer [20 mM Hepes-KOH (pH 7.4), 12 mM MgCl_2_, 100 mM KCl, 1% NP-40, 1 mM DTT, cycloheximide (100 μg/ml), and RNAsin (40 U/ml)] and incubated with end-over-end rotation for 1 hour at 4°C. Another set of Protein G Dynabeads was coupled to anti-FLAG antibody by incubating washed beads with anti-FLAG antibody (Sigma-Aldrich; catalog number: F1804) with end-over-end rotation for 1 hour at room temperature, followed by five washes with wash buffer. The supernatant of the precleared sample lysate was then transferred to the anti-FLAG–coupled Dynabeads and incubated by end-over-end rotation for 4 hours at 4°C. After incubation, the Dynabeads were washed three times with high salt buffer [20 mM Hepes-KOH (pH 7.4), 12 mM MgCl_2_, 350 mM KCl, 1% NP-40, 1 mM DTT, cycloheximide (100 μg/ml), and RNAsin (40 U/ml)], with the last wash rotating end-over-end for 20 min at 4°C, followed by two additional washes. After the final wash, 500 μl of TRIzol was added to the beads, the sample was vortexed and then incubated for 10 min on ice. The beads were then collected using a magnetic separation rack (Invitrogen, DynaMag-2 #12321D), and the supernatant transferred to a new tube. RNA isolation was performed using the Direct-zol RNA Microprep Kit (Zymo). RNA was reverse transcribed to cDNA using the Superscript IV cDNA Kit (Invitrogen), and elongase expression was measured using the Roche LightCycler with the LightCycler Master I Mastermix. Gene expression was normalized to the housekeeping gene RPL32.

### Gene expression analysis

High-throughput larval gene expression data from FlyAtlas (ERP024551) and modENCODE (SRP001696) were extracted from a comprehensive RNA sequencing tissue atlas that used published *Drosophila* datasets and homogenized the analysis workflow to make quantification comparable across studies (GSE117217). (*95*)

### Water loss assays

Prepupae were collected at 6 hours APF and removed from the side of the vial with a wet paintbrush. They were then transferred individually to a preweighed 0.2-ml PCR tube with the lid removed, and the tube plus prepupa was weighed again using a microbalance (Sartorius Lab Instruments, Sartorius Cubis MSE3.6P-000-DM). Water loss during metamorphosis was quantified under two conditions: (i) Tubes containing a single pupa were kept in a pipette tip box at the specified temperature and RH after pupariation. RH was measured using an RH probe, and high RH levels (∼95%) were achieved by adding wet tissues to the bottom of the pipette tip boxes. The weight of the tube plus pupa was measured every 24 hours after pupariation. (ii) Tubes containing a single pupa were transferred to the heat block of a PCR machine set to a constant temperature (25° to 29°C). The RH was not controlled but measured at between 15 and 20% using a Wireless Data Logger (T&D Corporation, RTR-574). The tube plus pupa was weighed every 24 hours after pupariation and, at 72 hours APF, dried at 60°C overnight and reweighed. Pupal wet and dry weights were calculated by subtracting the empty tube weight, and pupal water content was calculated by subtracting pupal dry weight at 72 hours APF from the pupal wet weight. For data shown in Fig. 5A, a loess curve was fitted to the data by genotype.

### Head eversion assays

Wandering third-instar larvae (at 96 hours after larval hatching) were transferred with a dry paintbrush to empty fly vials lacking food and placed at 25°C. Vials were then incubated in the ambient room environment (55 to 60% RH), in a box containing silica desiccant (low humidity, 25 to 30% RH) or in a box with wet paper towels (high humidity, 85 to 90% RH) as measured with an RH monitor (Hygrometer Testo 608-H1, Testo AG, Germany). Head eversion was scored after a 24-hour incubation. Images showing failed or completed head eversion were obtained from *EloHL*^Δ1^/+ pupae at 24 hours APF) immersed in mineral oil (Sigma-Aldrich, #M3516), mounted beneath a 1.5-mm coverslip, and imaged with a Leica MZ75 dissecting microscope using a Plan 1.0x objective and 1.6x zoom.

### Cryo-SIMS surface analysis

Pupae were collected with a paintbrush from the side of the vial at 48 hours APF. They were then attached to double-sided sticky tape (Hobbycraft) and removed from the larval cuticle (puparium) with watchmaker forceps. Pupae were then transferred to PBS, and a small incision was made at the anterior end of the pupal cuticle followed by a longitudinal cut with watchmaker scissors and the removal of all internal tissues. Empty pupal cuticles were then washed twice with PBS and twice with 150 mM ammonium formate. For cryogenic time-of-flight secondary ion mass spectrometry (cryo-ToF-SIMS), the empty pupal cuticles were mounted with either the internal or external surface facing uppermost on indium tin oxide (ITO)–coated glass coverslips (SPI Supplies, #6462-AB, 18 mm^2^, resistivity: 70 to 100 Ω) cut to size with a diamond knife. For cryo-OrbiSIMS, empty pupal cuticles were mounted on cleaned silicon wafers (Agar Scientific, #AGG3390-10).

Cryo-ToF-SIMS measurements were performed on a ToF-SIMS 5 instrument (IONTOF, GmbH) as previously described, using the SurfaceLab version 7.3 software (IONTOF, GmbH) (*78*). Samples on the ITO-coated glass slides were mounted on a cooling stage. The loadlock was flooded with nitrogen gas before cooling during pump-down. During the analysis, the sample was cooled with a copper cooling finger to ∼−110°C. Analyses were conducted using a 30-keV Bi_3_^+^ beam with a spot size of ∼1 μm and a current of 0.04 pA at a cycle time of 200 μs. Secondary ions were extracted with an extraction voltage of 1 kV, with delayed extraction mode activated. For measurements used to generate graphs, data were obtained from a field of view of 250 x 250 μm at a resolution of 128 x 128 pixels. The measurements shown are the sum of 20 scans. To compensate for sample charging, an electron flood gun was applied during analysis with an energy of 21 eV and a current of −10 μA. The total ion dose was ∼3.27 × 10^9^ ions/cm^2^. The mass resolution of the resulting spectra was ∼5000 at a mass/charge ratio (*m/z*) of 200. Spectral mass calibration was performed with reference to the known ions [C]^+^, [CH]^+^, [CH_2_]^+^, and [CH_3_]^+^. For measurements used to plot mass spectra, a total of 50 scans was acquired, with a total ion dose of ∼2.05 × 10^8^ ions/cm^2^. For quantification, the [M]^+•^ ion from hydrocarbons ranging in length from C29-C37 were summed. For these hydrocarbons, the presence and positive identification have been confirmed in pupal samples with GC-MS.

Cryo-OrbiSIMS measurements were performed on a Hybrid-SIMS instrument (IONTOF, GmbH) as previously described, using the SurfaceLab version 7.5 software (IONTOF, GmbH) (*78*). Samples were precooled in liquid nitrogen in a Leica VCM and loaded into the precooled loadlock with a Leica VCT. During the analysis, samples were kept at ∼−160°C. Analysis was performed with a 20-keV Ar_3500_^+^ GCIB with a spot size of ∼3 μm, a current of 13 pA, and a duty cycle of 10.15%. Secondary ions were extracted with an extraction voltage of 2 kV. Data were obtained from a field of view of 250 x 250 μm at a resolution of 70 x 70 pixels. The measurements shown are the sum of 50 scans. To compensate for sample charging, an electron flood gun was applied during analysis with an energy of 21 eV and a current of −20 μA. The total ion dose was ∼6 × 10^12^ ions/cm^2^. The mass resolution of the resulting spectra was ∼240,000 at *m/z* 200. Before data acquisition, the Orbitrap mass analyzer was calibrated against silver clusters using a silver standard. For hydrocarbon signal quantification, the [M+N]^+^ ion was used as previously described (*78*).

### Homologous gene identification and evolutionary analysis

To identify fatty acid elongases, TBLASTN (*96*) was used to search the reference genomes of the species in Fig. 4A using the protein sequences of the fatty acid elongase gene group from *D. melanogaster* (FBgg0000965) as queries, with a cutoff *e*-value of 1 × 10^−5^. Redundant hits in the same genomic regions were filtered out, and the sequences of all hits were extracted with extensions at both the 5′ and 3′ ends. Coding sequences were determined using Exonerate (*97*). To identify homologous regions and fatty acid elongases within the microsynteny, TBLASTN searches used as queries the protein sequences of the flanking genes from *D. melanogaster* (CG9467, CG8526, and FBXO11 on one side and CG42857, CG34302, and Teh1 on the other). The microsynteny was determined for all species in Fig. 4A except for *Scaptodrosophila lebanonensis* as a microsyntenic region could not be identified.

To determine the phylogenetic relationship between different elongase genes, we aligned the deduced amino acid sequences using MAFFT (*98*) and generated nucleotide sequence alignments based on the protein sequence alignment using PAL2NAL (*99*). A gene tree was inferred using IQ-TREE (*100*) with 1000 bootstrap replicates and the *Mortierella alpina* PUFA elongase (GenBank: AF206662.1) used as an outgroup. To investigate the selective pressures on different elongases within the microsynteny, we estimated nonsynonymous (*d*_N_) and synonymous (*d*_S_) substitution rates using the codon-based maximum likelihood method implemented in the CODEML program in PAML4.9 (*73*). Likelihood ratio tests using the chi-square approximation were used to compare nested models.

### Protein structural comparisons

The protein structures of EloHL and EloF were retrieved from the AlphaFold database (AlphaFold IDs AF-Q9VH59-F1-v4 and AF-Q9VH58-F1-v4, respectively) and compared through superposition using PyMOL (*101*).

### Data analysis

The production of graphs and statistical analysis were performed in RStudio version 4.2.3 (Posit Software, PBC, Boston, MA). Heatmaps were plotted using the superheat package (version 0.1.0), and all other graphs were plotted using ggplot2 (version 3.5.1). Statistical tests were performed using the packages lme (version 1.1-35.4), emmeans (version 1.10.1), and basic R. Statistical tests and results for each figure panel are listed in table S9.
