# Supplementary material for: Identification of a specialized lipid barrier for Drosophila metamorphosis
Source: Sci Adv. 2026 Nov 6;12(45):eadw6168. doi: 10.1126/sciadv.adw6168 (PMC13742154; doi:10.1126/sciadv.adw6168)
Supplement: Supplementary file 1 [file sciadv.adw6168_sm.pdf]

Science Advances
AAAS

Supplementary Materials for

Identification of a specialized lipid barrier for *Drosophila* metamorphosis

Lena Lampe *et al.*

Corresponding author: Alex P. Gould, alex.gould@crick.ac.uk

Sci. Adv. **12**, eadw6168 (2026)
DOI: 10.1126/sciadv.adw6168

The PDF file includes:

Figs. S1 to S6
Legends for tables S1 to S9

Other Supplementary Material for this manuscript includes the following:

Tables S1 to S9

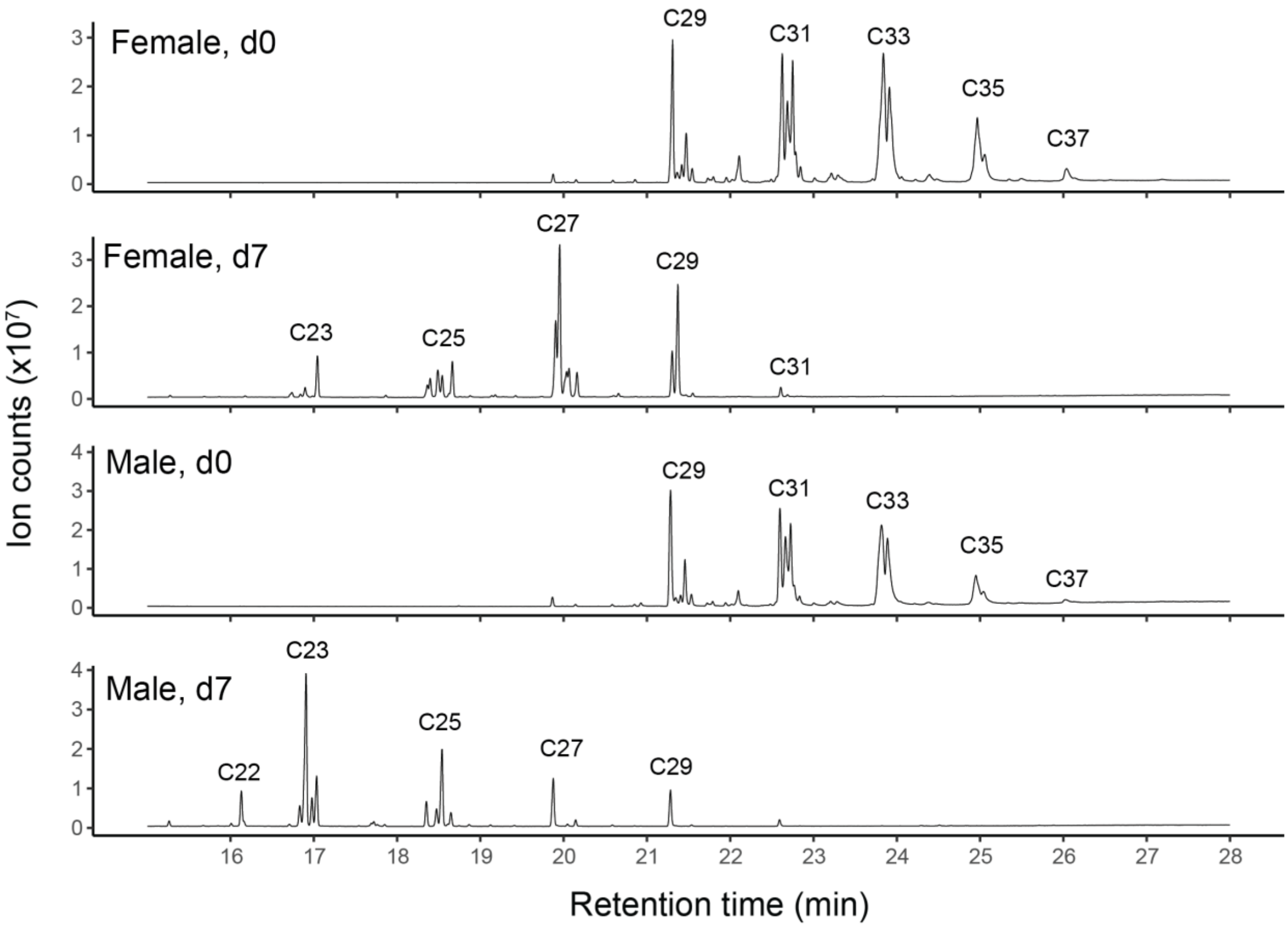

Fig S1.

Cuticular hydrocarbons of male and female flies at day 0 and 7, related to Figure 1.

Representative gas chromatograms from the HT-GC-MS analysis of newly emerged (day 0) and young (day 7) male and female flies. Cuticular hydrocarbon chain lengths (C23 to C37) are indicated.

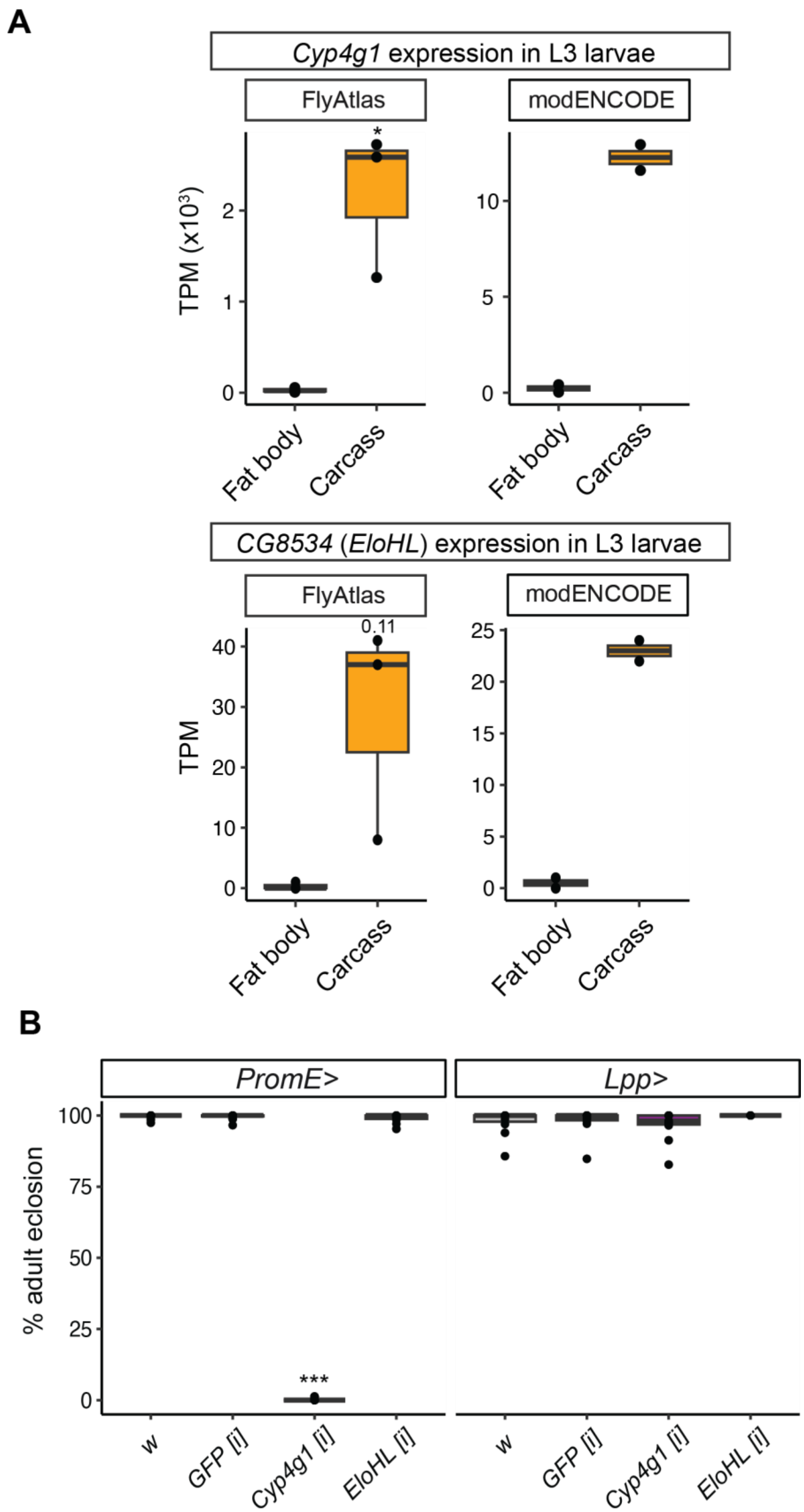
A
Cyp4g1 expression in L3 larvae
FlyAtlas
modENCODE
TPM (x10³)
Fat body
Carcass
*
CG8534 (EloHL) expression in L3 larvae
FlyAtlas
modENCODE
TPM
0.11
B
PromE>
Lpp>
% adult eclosion
***
w
GFP [i]
Cyp4g1 [i]
EloHL [i]

Fig. S2.

Expression and function of *Cyp4g1* and *EloHL* in the larval fat body and oenocytes, related to Figures 2 and 3. (**A**) Normalized expression (transcripts per million, TPM) data for *Cyp4g1* and *EloHL* in the larval fat body and carcass were extracted from two different published datasets — FlyAtlas (ERP024551; N=3) and modEncode (SRP001696; N=2). Expression was measured by RNAseq and re-processed in a standardized way (GSE117217). T-test was performed for studies with three replicates. (**B**) Adult eclosion after knockdown of *Cyp4g1* or *EloHL* in larval oenocytes (*PromE*>) or fat body (*Lpp*>). The environmental conditions are as in **Figure 2F** (N=3; n>3 fly vials).

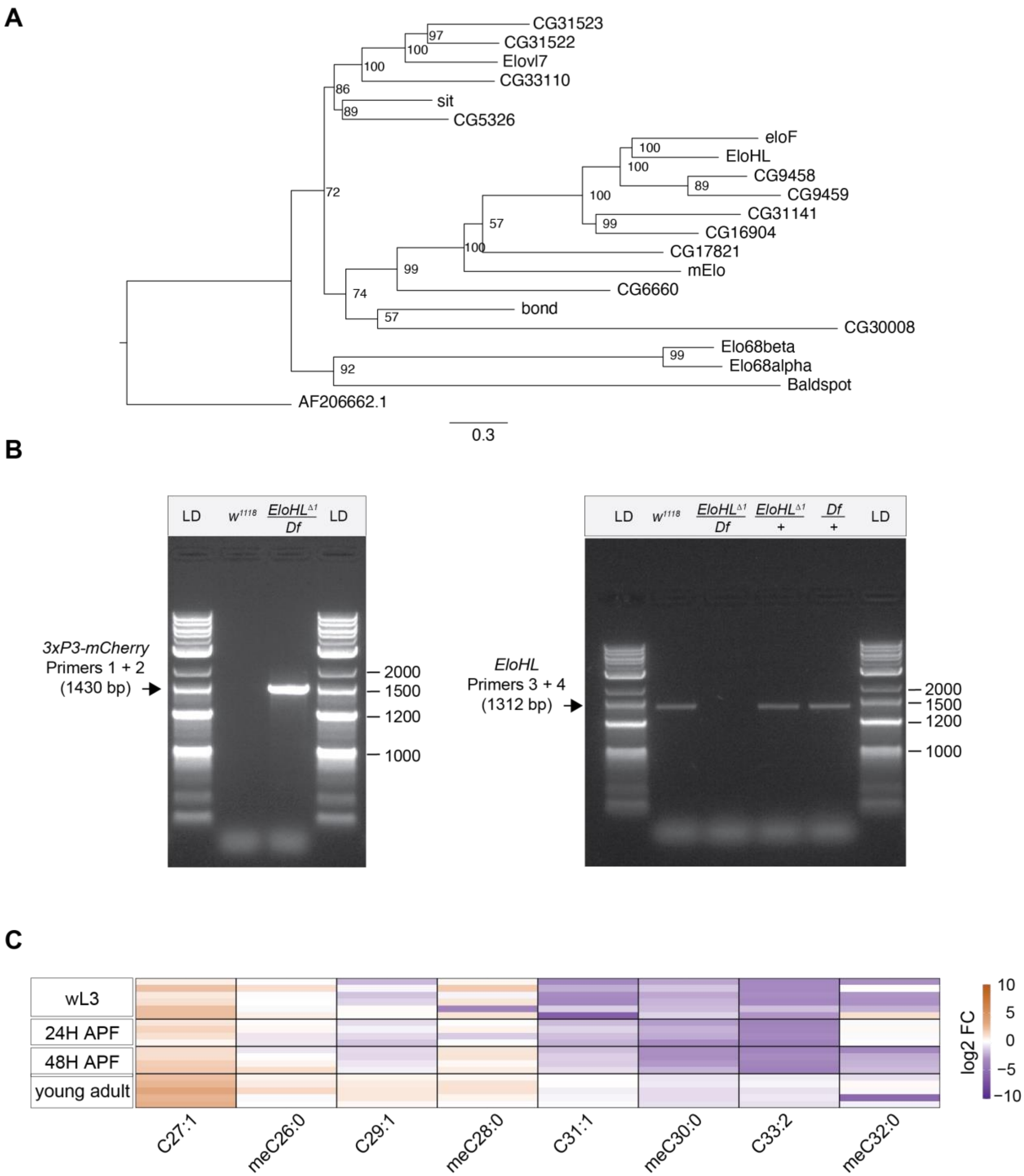
A
CG31523
CG31522
Elovl7
CG33110
sit
CG5326
eloF
EloHL
CG9458
CG9459
CG31141
CG16904
CG17821
mElo
CG6660
bond
CG30008
Elo68beta
Elo68alpha
Baldspot
AF206662.1
0.3
B
LD
w^{1118}
$EloHL^{\Delta1}$/Df
3xP3-mCherry
Primers 1 + 2
(1430 bp)
2000
1500
1200
1000
EloHL
Primers 3 + 4
(1312 bp)
$EloHL^{\Delta1}$/+
Df/+
C
wL3
24H APF
48H APF
young adult
C27:1
meC26:0
C29:1
meC28:0
C31:1
meC30:0
C33:2
meC32:0
log2 FC
10
5
0
−5
−10

Fig. S3.

Elongase phylogeny and analysis of the *EloHL*$^{\Delta1}$ mutation, related to Figure 3.

(A) Phylogeny of the 20 elongases identified in *Drosophila melanogaster*. The phylogenetic tree of fatty acid elongases in *D. melanogaster*. The gene tree was inferred using IQ-TREE with 1,000 bootstrap replicates and the *Mortierella alpina* long-chain polyunsaturated fatty acid elongation enzyme (GenBank: AF206662.1) was used as an outgroup.

(B) Left panel shows that integration of *3xP3::mCherry* at the *EloHL* locus gives a 1430 bp PCR product detected in *EloHL*$^{\Delta1}$*/Df(3R)Excel6155* but not in *w*^{1118} *iso31* control flies. Primer 1 is located outside the 5' homology arm and Primer 2 is located within *mCherry*. Right panel shows that the *EloHL* deletion lacks a 1312 bp PCR product, such that it is missing in *EloHL*$^{\Delta1}$*/Df(3R)Excel6155* but not in control (*w*^{1118} *iso31, Df(3R)Excel6155/+ or EloHL*$^{\Delta1}$ /+) flies. Primers 3 and 4 are located within exon 1 and exon 2 of *CG8534* respectively. The locations of Primers 1 to 4 and the strategy for replacing the open reading frame and 3'UTR of *EloHL* (*CG8534*) with a *3xP3::mCherry* cassette are shown in **Figure 3F**. NEB 1kbp Plus DNA ladder (LD) is indicated in both panels.

(C) Time course of HL hydrocarbons in *EloHL*$^{\Delta1}$ mutants at wandering L3 (wL3), at 24h and 48h after puparium formation (APF) and in young adults (0-6 hr after eclosion). Heatmap shows Log_2 fold changes of internal HCs of transheterozygotes (*EloHL*$^{\Delta1}$ */Df(3R)Excel6155*) relative to control heterozygotes (*EloHL*$^{\Delta1}$ /+ and *Df(3R) Excel6155/+*). The hydrocarbon molecules depicted are also shown in **Figure 3E and G**.

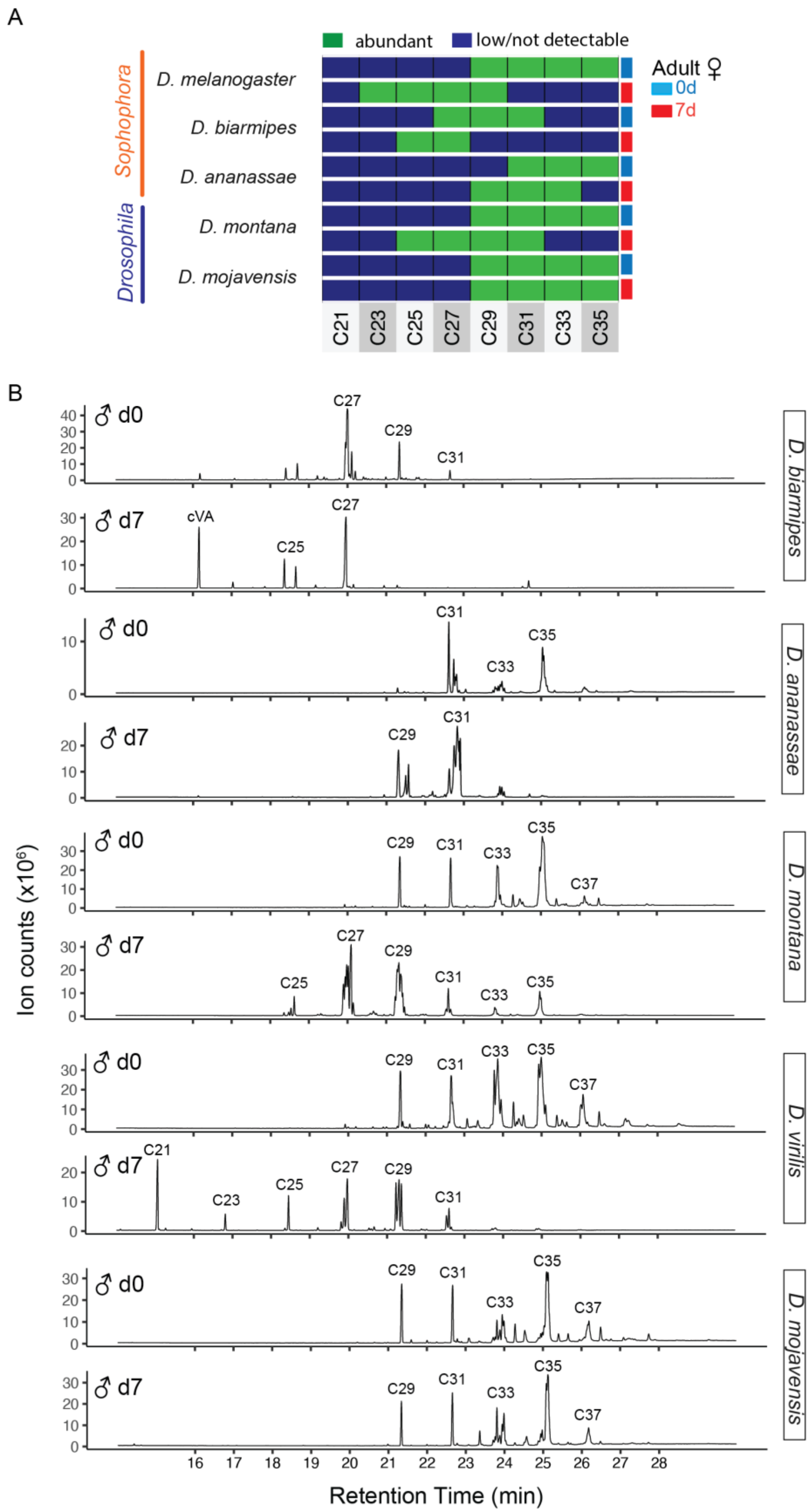
A
abundant
low/not detectable
Sophophora
Drosophila
D. melanogaster
D. biarmipes
D. ananassae
D. montana
D. mojavensis
Adult ♀
0d
7d
C21
C23
C25
C27
C29
C31
C33
C35
B
♂ d0
♂ d7
cVA
C37
D. biarmipes
D. ananassae
D. montana
D. virilis
D. mojavensis
Ion counts (x10^6)
Retention Time (min)

Fig. S4.
Many *Drosophila* species transition from hyper-long to shorter hydrocarbons, related to Figure 4.

(**A**) Abundant cuticular hydrocarbons of newly eclosed (day 0, blue) and one week old (day 7, red) female flies from the subgenera *Sophophora* (*D. melanogaster, D. biarmipes, D. ananassae*) and *Drosophila* (*D. montana, D. mojavensis*). Abundant (green) or low/not detected (blue) hydrocarbons are indicated according to length (C21 to C35) but pooling alkanes, methyl-branched alkanes, monoenes, and dienes.
(**B**) Representative gas chromatograms of cuticular hydrocarbons from newly eclosed (day 0, blue) and one week old (day 7, red) male flies of the indicated species. Carbon-chain lengths are indicated. These data were analyzed for **Figure 4A**.

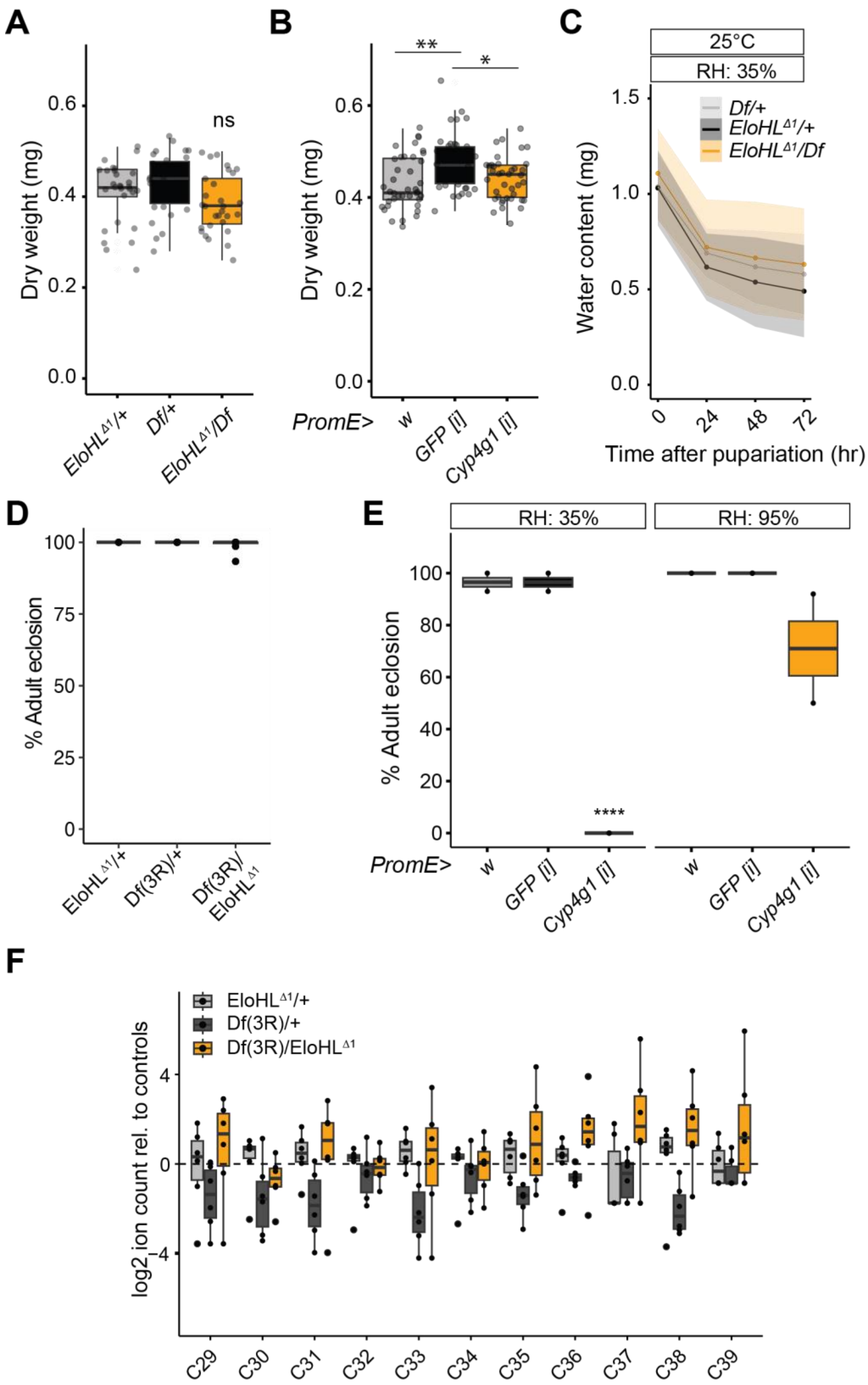

A
B
C
Dry weight (mg)
0.0
0.2
0.4
0.6
ns
EloHL^Δ1/+
Df/+
EloHL^Δ1/Df
**
*
PromE>
w
GFP [i]
Cyp4g1 [i]
25°C
RH: 35%
Water content (mg)
Df/+
EloHL^Δ1/+
EloHL^Δ1/Df
0.0
0.5
1.0
1.5
0
24
48
72
Time after pupariation (hr)
D
% Adult eclosion
0
25
50
75
100
EloHL^Δ1/+
Df(3R)/+
Df(3R)/ EloHL^Δ1
E
RH: 35%
RH: 95%
0
20
40
60
80
100
****
F
EloHL^Δ1/+
Df(3R)/+
Df(3R)/EloHL^Δ1
log2 ion count rel. to controls
4
0
-4
C29
C30
C31
C32
C33
C34
C35
C36
C37
C38
C39

Fig. S5.

***EloHL*$^{\Delta1}$ and oenocyte *Cyp4g1 [i]* dry weights, water content, eclosion rates and pupal cuticle hydrocarbon profiles, related to Figure 5.**

(A) Pupal dry weights (mg) of *EloHL*$^{\Delta1}$*/Df(3R)Excel6155* pupa and heterozygous controls (*EloHL*$^{\Delta1}$*/+; Df/+*) at dark pharate stage. N=3, n = 10.
(B) Pupal dry weights (mg) of oenocyte-specific *Cyp4g1* knockdown (*PromE>Cyp4g1 [i]*) and control (*PromE/+ or PromE>GFP[i]*) animals at dark pharate stage. N=2, n ~ 20.
(C) Pupal water content (mg) versus time after pupariation for *EloHL*$^{\Delta1}$*/Df(3R)Excel6155* mutants and heterozygous controls (*EloHL*$^{\Delta1}$*/+; Df/+*) at low relative humidity (35% RH) and 25°C. N=3; n=10.
(D) *EloHL* is not required for adult eclosion under standard laboratory conditions. *EloHL*$^{\Delta1}$*/Df(3R)Excel6155* and heterozygous controls (*EloHL*$^{\Delta1}$*/+ or Df(3R)Excel6155/+*) show almost complete adult eclosion at 25°C, 60% relative humidity. n = 3-8 vials.
(E) High relative humidity (95% RH)) rescues adult eclosion of oenocyte-specific *Cyp4g1* knockdowns. *PromE>Cyp4g1[i]*, *PromE/+* and *PromE>GFP [i]* control animals were raised at 25°C with 35% or 95% relative humidity (RH) from pupariation onwards. Statistical significance indicated by ****(p<0.00005). N=2, n = 10.
(F) Hydrocarbons on the external surface of the pupal cuticle at 48h APF are not EloHL dependent. Surface-specific cryogenic mass spectrometry imaging (cryo-OrbiSIMS) was used to measure HL hydrocarbons. Hydrocarbons of the same carbon length were pooled for analysis. Genotypes are *EloHL*$^{\Delta1}$*/Df(3R)Excel6155* transheterozygotes and control heterozygotes (*EloHL*$^{\Delta1}$*/+* or *Df(3R)Excel6155 /+*). n = 8 pupal cuticles.

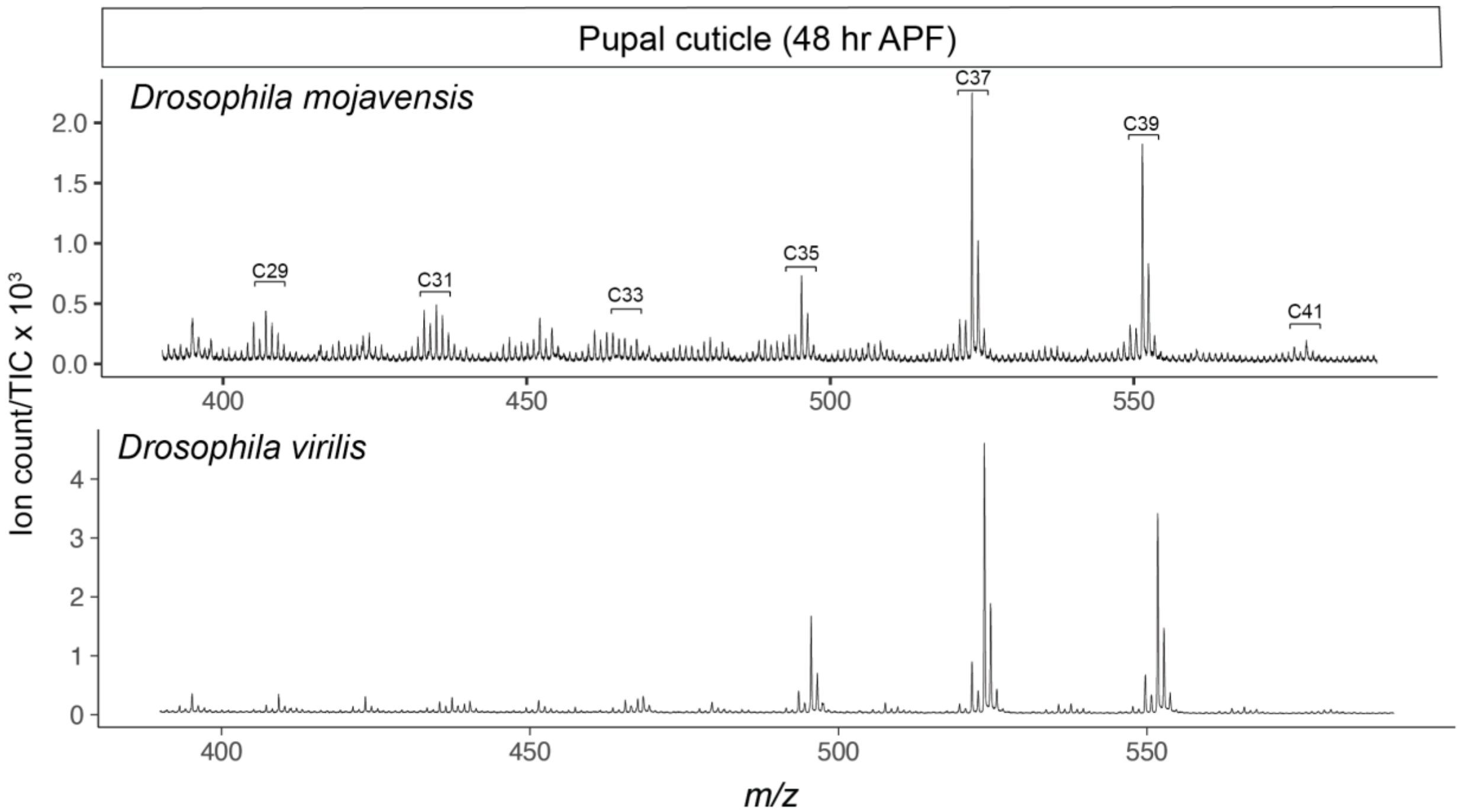

Fig. S6.

Hyper-long hydrocarbons on the pupal cuticle of *Drosophila mojavensis* and *Drosophila virilis,* related to Figure 6. Representative cryo-ToF-SIMS spectra from the outer surface of the pupal cuticle at 48h after puparium formation (APF). Hydrocarbons from C29 to C41 are indicated. The Y axis indicates ion count relative to the total ion count (TIC).

Table S1.
Quantification of cuticular surface hydrocarbons for larvae (wandering L3 stage), pupae (48h APF), and male and female adults (0h, 6h, 24h, 3 days and 7 days after eclosion). Data used in **Figure 1A**.

Table S2.
Quantification of three independent replicates of internal hydrocarbons extracted from larvae (wandering L3 stage) or newly eclosed adults (0h after eclosion). Data used in **Figure 2A**.

Table S3.
Quantification of internal hydrocarbons extracted from the carcass, fat body or gut of wandering L3 larval tissues. Data used in **Figure 2B**. ND = not detected

Table S4.
Quantification of internal hydrocarbons extracted from wandering L3 larvae. Data used in **Figure 3E and 3G**.

Table S5.
Quantification of internal hydrocarbons from whole wL3 larvae, 24h APF pupae, 48h APF pupae, and newly eclosed adults. Data used in **Figure S3C.**

Table S6.
PAML analysis of the four phylogenetic models (A to D). Data used in **Figure 4E**.

Table S7.
Quantification of hydrocarbons extracted from the larval cuticle at 12h APF. Data used in **Figure 5I**.

Table S8.
Primer sequences used in this study.

Table S9.
Statistical analyses and p values for each Figure panel.
